# Coordinated Optimization of Visual Cortical Maps (II) Numerical Studies

**DOI:** 10.1371/journal.pcbi.1002756

**Published:** 2012-11-08

**Authors:** Lars Reichl, Dominik Heide, Siegrid Löwel, Justin C. Crowley, Matthias Kaschube, Fred Wolf

**Affiliations:** 1 Max-Planck-Institute for Dynamics and Self-Organization, Göttingen, Germany; 2 Bernstein Center for Computational Neuroscience, Göttingen, Germany; 3 Bernstein Focus Neurotechnology, Göttingen, Germany; 4 Faculty of Physics, Georg-August University, Göttingen, Germany; 5 Frankfurt Institute of Advanced Studies, Frankfurt, Germany; 6 School of Biology, Georg-August University, Göttingen, Germany; 7 Carnegie Mellon University, Department of Biological Sciences, Pittsburgh, Pennsylvania, United States of America; 8 Physics Department and Lewis-Sigler Institute, Princeton University, Princeton, New Jersey, United States of America; 9 Kavli Institute for Theoretical Physics, University of California, Santa Barbara, California, United States of America; Indiana University, United States of America

## Abstract

In the juvenile brain, the synaptic architecture of the visual cortex remains in a state of flux for months after the natural onset of vision and the initial emergence of feature selectivity in visual cortical neurons. It is an attractive hypothesis that visual cortical architecture is shaped during this extended period of juvenile plasticity by the coordinated optimization of multiple visual cortical maps such as orientation preference (OP), ocular dominance (OD), spatial frequency, or direction preference. In part (I) of this study we introduced a class of analytically tractable coordinated optimization models and solved representative examples, in which a spatially complex organization of the OP map is induced by interactions between the maps. We found that these solutions near symmetry breaking threshold predict a highly ordered map layout. Here we examine the time course of the convergence towards attractor states and optima of these models. In particular, we determine the timescales on which map optimization takes place and how these timescales can be compared to those of visual cortical development and plasticity. We also assess whether our models exhibit biologically more realistic, spatially irregular solutions at a finite distance from threshold, when the spatial periodicities of the two maps are detuned and when considering more than 2 feature dimensions. We show that, although maps typically undergo substantial rearrangement, no other solutions than pinwheel crystals and stripes dominate in the emerging layouts. Pinwheel crystallization takes place on a rather short timescale and can also occur for detuned wavelengths of different maps. Our numerical results thus support the view that neither minimal energy states nor intermediate transient states of our coordinated optimization models successfully explain the architecture of the visual cortex. We discuss several alternative scenarios that may improve the agreement between model solutions and biological observations.

## Author Summary

Neurons in the visual cortex of carnivores, primates and their close relatives form spatial representations or maps of multiple stimulus features. In part (I) of this study we theoretically predicted maps that are optima of a variety of optimization principles. When analyzing the joint optimization of two interacting maps we showed that for different optimization principles the resulting optima show a stereotyped, spatially perfectly periodic layout. Experimental maps, however, are much more irregular. In particular, in case of orientation columns it was found that different species show apparently species invariant statistics of point defects, so-called pinwheels. In this paper, we numerically investigate whether the spatial features of the stereotyped optima described in part (I) are expressed on biologically relevant timescales and whether other, spatially irregular, long-living states emerge that better reproduce the experimentally observed statistical properties of orientation maps. Moreover, we explore whether the coordinated optimization of more than two maps can lead to spatially irregular optima.

## Introduction

In the primary visual cortex of primates and carnivores, functional architecture can be characterized by maps of various stimulus features such as orientation preference (OP), ocular dominance (OD), spatial frequency, or direction preference [1]–[21]. Many attempts have been made to explain and understand the spatial organization of these maps as optima of specific energy functionals the brain minimizes either during development or on evolutionary timescales [22]–[38]. In part (I) of this study we presented an analytical approach to study the coordinated optimization of interacting pairs of visual cortical maps where maps are described by real and complex valued order parameter fields [39]. We used symmetry considerations to derive a classification and parametrization of conceivable inter-map coupling energies and identified a representative set of inter-map coupling terms: a gradient-type and a product-type coupling energy which both can enter with different power in the dynamics. Examining this set of inter-map coupling energies was further motivated by the experimentally observed geometric relationships between cortical maps [5], [7], [15], [19], [26], [40], [41]. We examined the impact of these coupling energies in a system of coupled Swift-Hohenberg equations. These were constructed such that without coupling stripe patterns emerge for the complex valued order parameter field. We found that these types of inter-map coupling energies can induce the formation of defect structures, so-called pinwheels, in the complex order parameter field describing the OP map. For solutions that can become optima of the model, pinwheels are arranged on regular periodic lattices such as rhombic pinwheel crystals (rPWCs) or hexagonal pinwheel crystals (hPWCs). These analyses focused on the optimization of a single pair of feature maps in which the complex valued map represented the OP map and the real map the OD map. For this case we presented a complete characterization of the stable OP and OD patterns, stripe-like solutions, rhombic and hexagonal crystalline patterns predicted by the coordinated optimization models. In all analyzed models pinwheel crystallization required a substantial bias in the response properties of the co-evolving real-valued map.

**Figure 1:**
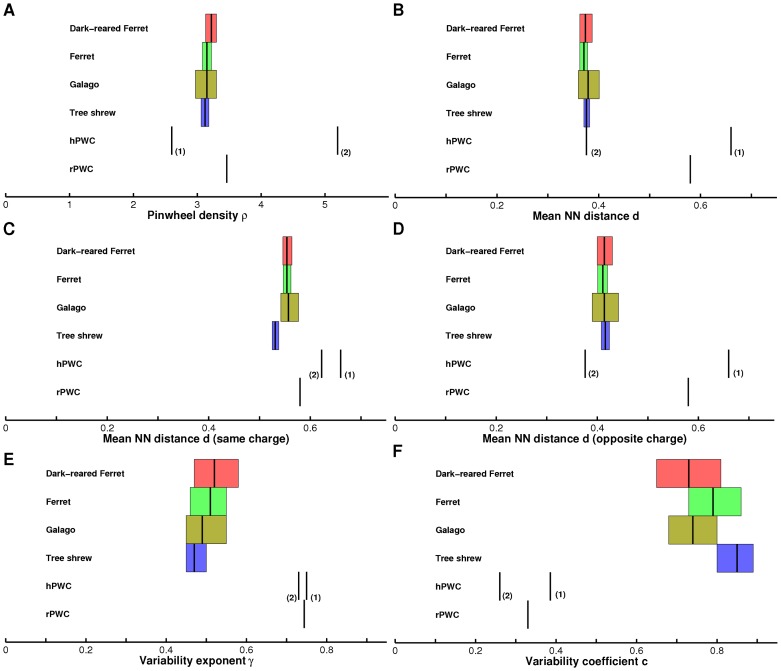
Comparison of pinwheel statistics for galago, ferret, dark-reared ferret, tree shrew, and PWC solutions. **A** Pinwheel density, **B,C,D** Pinwheel nearest neighbor statistics. **E,F** Pinwheel density variability exponent **E** and coefficient **F**. The two hPWC solutions correspond to the ‘Braitenberg’ (1) and ‘Ipsi-center’ (2) PWC obtained in part (I). Bars are centered around the population mean and span the 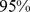 confidence intervals. Animal data from [42].

The pinwheel crystals we obtained, although beautiful and easy to characterize, qualitatively deviate from the spatially irregular layout observed for OP maps in the visual cortex [2]–[4]. Large scale empirical studies of the arrangement of pinwheel positions and spatial densities in the visual cortex of four species widely separated in mammalian evolution recently showed that orientation maps although spatially irregular precisely conform with apparently species insensitive quantitative layout rules [42], [43]. In particular, it was found that not only the mean density of pinwheels but also number fluctuations over a wide range of spatial scales and local next neighbor arrangements within individual hypercolumns agree across species with an accuracy in the range of a few percent [42], [43], see also Fig. 1. In contrast to the large variability of local map layouts in experimentally observed maps [1]–[21], the pinwheel crystals found in the coordinated optimization models introduced in part (I) show a regular and stereotyped structure. Quantitatively, all PWC solutions that we found exhibit a large pinwheel density of about 3.5 or even 5.2 pinwheels per hypercolumn. For experimental OP maps the average pinwheel density was found to be between 3.1 and 3.2 and statistical indistinguishable from the mathematical constant 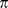 up to a precision of 2% [42], [44], [45]. Our previous analytical results thus raise the question of whether and how our coordinated optimization models can be reconciled with the experimentally observed layout rules of orientation maps.

From a biological perspective, one might suspect that the crystalline layouts of local minima and optima results from the restrictions of the applied perturbation method which allowed us to study optima analytically but might be biased towards particular solution classes. Furthermore, results might change substantially if one would consider the coordinated optimization of more than two feature maps. Examining this aspect is also demanded because of the presence of multiple feature maps in the visual cortex of primates and carnivores. Furthermore geometrical rules coordinating map layout might in general be the harder to satisfy the more maps are simultaneously optimized. Finally, when studying optima predicted by a particular optimization principle we disregarded transient states that could in principle dominate developmental optimization on biologically relevant timescales. Such transient solutions are expected to be more irregular than the final attractor states. Analytical results were obtained using a perturbative treatment close to the pattern forming threshold. This perturbative treatment, however, gives no information on the speed with which singularities will crystallize into highly ordered arrays. It is conceivable that this process may occur on very long timescales. If this was the case, developmental optimization may lead to long-lived spatially irregular states that are transients towards regular patterns that would be reached after very long times or potentially never. To assess this possibility it is critical to examine model predictions over a wide range of timescales and compare biological developmental phases to different stages in numerical model simulations. In the current study we propose a systematic procedure for such comparisons that is based on a wide array of development experiments and theoretical analyses.

Numerical studies complementing the analyses presented in part (I) are also demanded for various theoretical reasons. In part (I) we showed that one can neglect the backreaction of the OP map onto the OD map if the OD map is ‘dominant’ i.e. its amplitude is much larger than that of the OP map. This can be achieved for a sufficiently small ratio of their distances to threshold. This finding raises questions that cannot be easily addressed perturbatively. Do the observed local minima and optima of the optimization principles persist when taking the backreaction into account or when considering map formation further from the pattern formation threshold? Besides the influence of the backreaction, the full dynamical system receives additional corrections. There are higher order corrections to the uncoupled amplitude equations which can become important for finite bifurcation parameters but were neglected in part (I) [39]. In part (I) of this study we also assumed equal periodicities of the two interacting maps. Systematic differences of OD and OP wavelengths have been observed for instance in macaque monkey visual cortex [7], [46]. In case of cat visual cortex different OP and OD wavelength have been observed within the same animal [47] although the average wavelength of the OD and OP pattern appears similar on average [48], [49]. Experiments suggest that the different periodicities in the layout of OP and OD maps can have an impact on the map layout [7], [48], [49]. It is thus also interesting to explore whether and how a detuning of typical periodicities affects optimal layouts and whether it can lead to spatially irregular maps.

To assess these issues we generalized the field dynamics to describe the coordinated optimization of coupled complex valued and several real valued scalar fields. From a practical point of view, the analyzed phase diagrams and pattern properties indicate that the higher order gradient-type coupling energy is the simplest and most convenient choice for constructing models that reflect the correlations of map layouts in the visual cortex. For this coupling, intersection angle statistics are reproduced well, pinwheels can be stabilized, and pattern collapse cannot occur. In the current study we thus numerically analyzed the dynamics of coordinated optimization focusing on the high order gradient-type inter-map coupling energy. We use a fully implicit integrator based on the Crank-Nicolson scheme and a Newton-Krylow solver. In numerical simulations we characterize the kinetics and conditions for pinwheel crystallization and the creation of pinwheels from a pinwheel-free initial pattern. We assessed layout parameters of OP maps throughout all stages of optimization. To aid comparison with developmental timescales all results are represented with time normalized to the time required for maturation of orientation selectivity. Creation of pinwheels from a pinwheel-free initial pattern is a sufficient although not a necessary criterion for systems in which a pinwheel-rich state is energetically favored. As we point out this criterion can be easily assessed in models of arbitrary complexity that otherwise evade analytical treatment. We further explored the impact of inter-map wavelength differences, as observed in certain species, on the structure of the resulting solutions. Finally, we extended the models to explore the coordinated optimization of more than two feature maps. To examine whether the observed quantitative properties can be reproduced in models for the coordinated optimization of maps we calculated various pinwheel statistics during optimization. We find that spatially irregular patterns decay relatively fast into locally crystalline arrays. Further long-term rearrangement mainly leads to the emergence of long-range spatial alignment of local crystalline arrangements. We showed that our previous finding that OD stripes are unable to stabilize pinwheels generalizes to the case of detuned wavelengths. The observation that the coordinated optimization of two interacting maps leads to spatially perfectly periodic optima is also robust to detuned typical wavelengths and to the inclusion of more than two feature maps. Our results suggest that the coordinated optimization of multiple maps that would in isolation exhibit spatially perfectly periodic optimal layouts on its own does not offer a simple explanation for the experimentally observed spatially irregular design of OP maps in the visual cortex and its quantitative aspects. We consider alternative scenarios and propose ways to incorporate inter-map relations and joint optimization in models in which the optimal OP map layout is intrinsically irregular already for vanishing inter-map coupling.

## Results

### Dynamical systems approach

We model the response properties of neuronal populations in the visual cortex by two-dimensional scalar order parameter fields which are either complex valued or real valued [50]–[52]. We consider inter-map coupling between a complex valued map 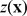 and one or several real valued maps 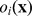. The complex valued field 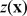 can for instance describe OP or direction preference of a neuron located at position 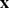. A real valued field 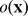 can describe for instance OD or spatial frequency preference. Although we consider a model for the coordinated optimization of general real and complex valued order parameter fields we view 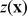 as the field of OP throughout this article to aid comparison to the biologically observed patterns. In this case, the pattern of preferred stimulus orientation 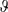 is obtained by
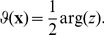
(1)The modulus 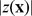 is a measure of orientation selectivity at cortical location 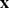.

OP maps are characterized by so-called *pinwheels*, regions in which columns preferring all possible orientations are organized around a common center in a radial fashion [50], [53]–[55]. The centers of pinwheels are point discontinuities of the field 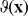 where the mean orientation preference of nearby columns changes by 90 degrees. Pinwheels can be characterized by a topological charge 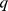 which indicates in particular whether the orientation preference increases clockwise or counterclockwise around the pinwheel center,
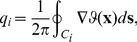
(2)where 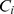 is a closed curve around a single pinwheel center at 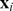. Since 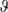 is a cyclic variable in the interval 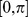 and up to isolated points is a continuous function of 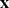, 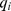 can only have values
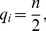
(3)where 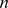 is an integer number [56]. If its absolute value 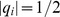, each orientation is represented only once in the vicinity of a pinwheel center. In experiments, only pinwheels with a topological charge of 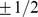 have been observed, which are simple zeros of the field 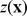.

In case of a single real valued map 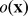 the field can be considered as the field of OD, where 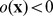 indicates ipsilateral eye dominance and 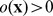 contralateral eye dominance of the neuron located at position 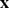. The magnitude indicates the strength of the eye dominance and thus the zeros of the field corresponding to the borders of OD domains.

If visual cortical maps are described by optima of an energy functional 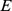, a formal time evolution of these maps that represents the gradient descent of this energy functional can be used to obtain predicted map layouts. The field dynamics thus takes the form
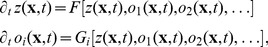
(4)where 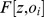 and 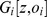 are nonlinear operators given by 
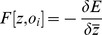
, 
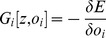
. The system then relaxes towards the minima of the energy 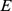. The convergence of this dynamics towards an attractor is assumed to represent the process of maturation and optimization of the cortical circuitry. Various biologically detailed models can be cast into the form of Eq. (4) [22], [52], [57].

**Figure 2:**
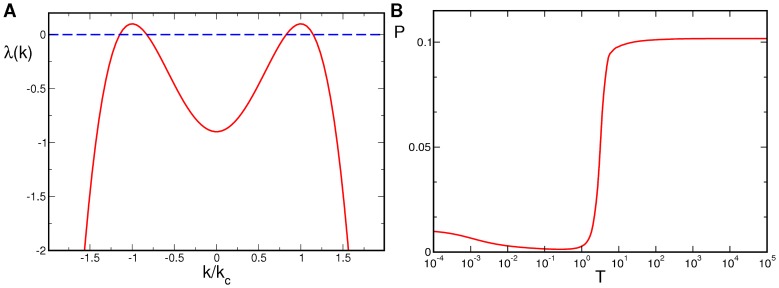
Swift-Hohenberg equation. **A** Cross section through the spectrum 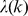 of the Swift-Hohenberg operator Eq. (7), 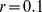. **B** Time evolution of the Power, Eq. (8), for spatial white noise initial conditions.

To dissect the impact of inter-map coupling interactions we split the energy functional 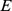 into single field and interaction components 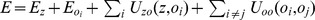. All visual cortical maps are arranged in roughly repetitive patterns of a typical wavelength 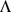 that may be different for different maps. We chose 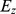 to obtain, in the absence of coupling, a well studied model reproducing the emergence of a typical wavelength by a pattern forming instability, the Swift-Hohenberg model [58], [59]. This model has been characterized comprehensively in the pattern formation literature and mimics the behavior of for instance the continuous Elastic Network or the Kohonen model for orientation selectivity (see [22]). We note that many other pattern forming systems occurring in different physical, chemical, and biological contexts (see for instance [60]–[63]) have been cast into a dynamics of the same form. Its dynamics in case of the OP map is of the form
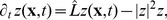
(5)with the linear Swift-Hohenberg operator
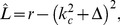
(6)

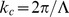
, and 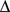 the Laplace operator. In Fourier representation, 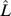 is diagonal with the spectrum
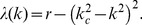
(7)The spectrum exhibits a maximum at 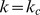, see Fig. 2**A**. For 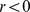, all modes are damped since 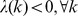 and only the homogeneous state 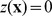 is stable. This is no longer the case for 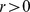 when modes on the *critical circle* 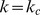 acquire a positive growth rate and grow, resulting in patterns with a typical wavelength 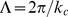. This model exhibits a supercritical bifurcation where the homogeneous state looses its stability and spatial pattern emerge.

While the linear part of the dynamics establishes a typical wavelength, the nonlinear term in the dynamics leads to the selection of the final pattern [64], [65]. Considering the time evolution following Eq. (5) initialized with a random OP map and low selectivity (small 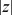) several different stages of the dynamics can be distinguished. The linear part forces modes on the critical circle to grow with rate 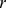 while strongly suppressing modes off the critical circle when starting from small amplitude white noise initial conditions, see Fig. 2**A**. The OP map becomes more ordered in this linear phase as one dominant wavelength emerges. The total power of the field is given by
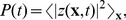
(8)where 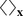 denotes spatial average. The time dependence of the power reflects the different growth rates among modes. The time evolution of the power is depicted in Fig. 2**B**. Initially, the power decreases slightly due to the suppression of modes outside the circle of positive growth rate. At 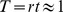 there is a rapid increase followed by the saturation of the power. The amplitudes of the Fourier modes reach their stationary values and 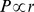. At this stage of the evolution the influence of the nonlinear part becomes comparable to that of the linear part. Once the modes saturate the phase of nonlinear competition between the active modes along with a reorganization of the structure of the OP map starts. The competition between active modes leads to pattern selection i.e. the convergence toward one of the in principle infinitely many periodic and aperiodic fixed points of the evolution equations. The final pattern then consists of distinct modes in Fourier space [59], [64]. Once the active modes are selected a relaxation of their phases takes place. These stages thus represent an initial process of selectivity maturation and a process of convergence to a stationary layout. This suggests to compare the first stage to the biological developmental period in which neurons reach adult-like levels of orientation selectivity and the later convergence stage to the following period of developmental juvenile plasticity e.g. until the closure of the developmental critical periods. To aid a detailed comparison we are presenting all maps and layout parameters as a function of time. In such displays time, during gradient descent optimization, is represented in two different ways. Firstly, following conventions in the pattern formation literature, time is rescaled with the largest growth rate 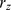 of the OP map, 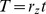. Secondly, to aid comparison with biological observations, we also graph all calculated layout properties as a function of ‘developmental time’ 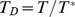 where 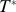 is the time for which the OP power reaches its peak value or, if there is no peak in the OP power, reaches 90% of its final value. In these units 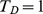 represents the time when orientation selectivity is essentially mature and later times correspond to subsequent convergence processes.

Inter-map coupling can influence the time evolution on all stages of the development depending on whether this coupling affects only the nonlinear part or also the linear one. When incorporating additional maps into the system in all cases we rescaled the dynamics by the bifurcation parameter of the OP map i.e. 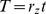. The coupled dynamics we considered is of the form
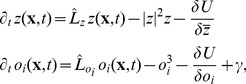
(9)where 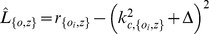, and 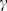 is a constant. To account for the differences in the dominant wavelengths of the patterns we chose two typical wavelengths 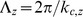 and 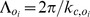. In the sections ‘Final states’ and ‘Kinetics of pinwheel crystallization’ we assume 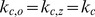 i.e. the Fourier components of the emerging pattern are located on a common circle. In the subsequent sections we also consider a potential detuning of the typical wavelength. The dynamics of 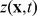 and 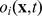 are coupled by interaction terms which can be derived from a coupling energy 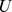. Many optimization models of the form presented in Eq. (4) have been studied [22]–[38]. The concrete dynamics in Eq. (9) is the simplest which in the uncoupled case leads to pinwheel-free OP stripe patterns and to a stripe-like or patchy layout of the co-evolving real valued fields.

As revealed by the symmetry-based classification of coupling energies
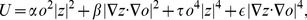
(10)parametrizes a representative family of biologically plausible coupling energies for a single real valued map 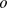, see part (I), [39].

The numerical integration scheme to solve Eq. (9) is detailed in the Methods part. For numerical analysis we focused on the high order gradient-type inter-map coupling energy. This energy can reproduce all qualitative relationships found between OP and OD maps, does not suffer from potential OP map suppression, and leads to a relatively simple phase diagram for two interacting maps near threshold.

### Final states

**Figure 3:**
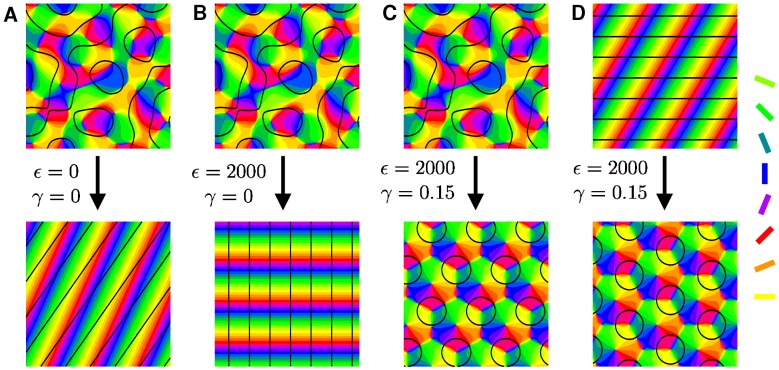
Pinwheel annihilation, preservation, and generation in numerical simulations for different strengths of inter-map coupling 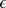 and OD bias 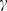. Color code of OP map with zero contours of OD map superimposed. **A** 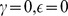 **B** 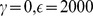 **C** and **D** 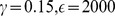. Initial conditions identical in **A**–**C**, 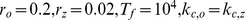.

In part (I) we calculated phase diagrams for different inter-map coupling energies [39]. In all cases, hexagonal PWCs can be stabilized only in case of OD hexagons. We tested these results numerically. Numerical simulations of the dynamics Eq. (9) with the coupling energy
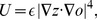
(11)are shown in Fig. 3. All remaining inter-map coupling energies in Eq. (10) are assumed to be zero. Initial conditions for the OD map were chosen as spatially irregular patterns or stripe patterns with saturated power plus Gaussian white noise. Initial conditions for the OP map are either pinwheel-free OP stripes or band-pass filtered Gaussian white noise for which the average pinwheel density is bounded from below by the constant 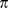 [22]. The initial conditions and final states are shown for different bias terms 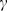 and inter-map coupling strengths 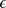. We observed that for a substantial contralateral bias and above a critical inter-map coupling pinwheels are preserved for all times or are generated if the initial condition is pinwheel-free. Without a contralateral bias the final states were pinwheel-free stripe solutions irrespective of the strength of the inter-map coupling.

### Kinetics of pinwheel crystallization

**Figure 4:**
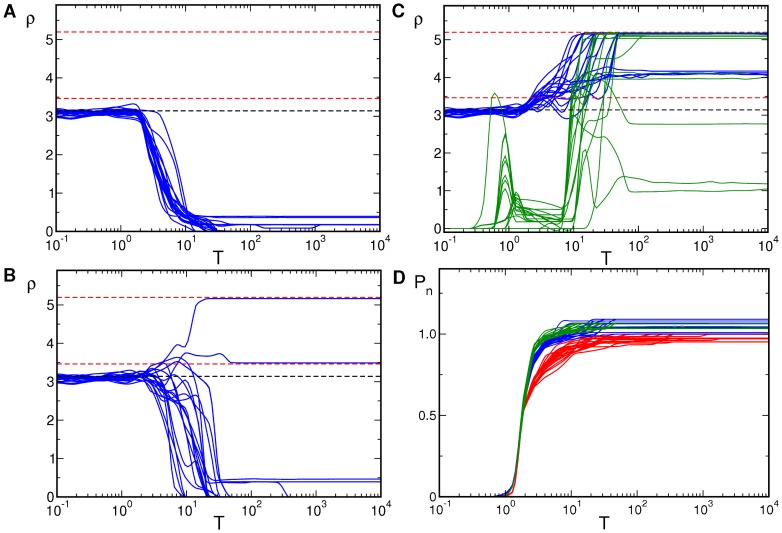
Time evolution of the pinwheel density, 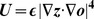, 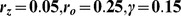. For each parameter set **A**–**C** simulations in blue started from an identical set of 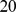 initial conditions. Red dashed lines: 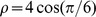 and 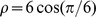, black dashed line: 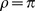. **A** 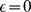 **B** 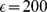 **C** 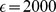. **D** Normalized power of OP map, 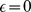 (red), 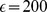 (blue), and 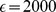 (green). In green **C**: OD and OP stripes as initial conditions. Parameters: 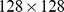 mesh, 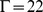.

To characterize the process of pinwheel annihilation, preservation, and creation during progressive map optimization we calculated the pinwheel density as well as various other pinwheel statistics (see Methods) during the convergence of patterns to attractor states. The time evolution of the pinwheel density is shown in Fig. 4. Initial conditions for the OD map were chosen as hexagonal patterns plus Gaussian white noise. Initial conditions for the OP map are either pinwheel-free OP stripes or band-pass filtered Gaussian white noise. Note the logarithmic time scales. Pinwheel densities rapidly diverge from values near 3.1 as soon as the map exhibits substantial power 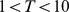. In the uncoupled case (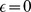) most of the patterns decayed into a stripe solution and their pinwheel density dropped to a value near zero. At small coupling strengths (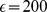) the pinwheel density converged either to zero (stripes), to values near 3.5 for the rPWC (see Fig. S6 in part (I), [39]), or to approximately 5.2 for the contra-center hPWC (see Fig. S7 in part (I), [39]). At high inter-map coupling (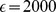) pinwheel free stripe patterns formed neither from pinwheel rich nor from pinwheel free initial conditions. In this regime the dominant layout was the contra-center hPWC. When starting from OD and OP stripes, see Fig. 4**C** (green lines), the random orientation between the stripes first evolved towards a perpendicular orientation (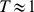). This lead to a transient increase in the pinwheel density. At the time (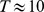) where the OD stripes dissolve towards OD hexagons hPWC solutions formed and the pinwheel density reached its final value.

**Figure 5:**
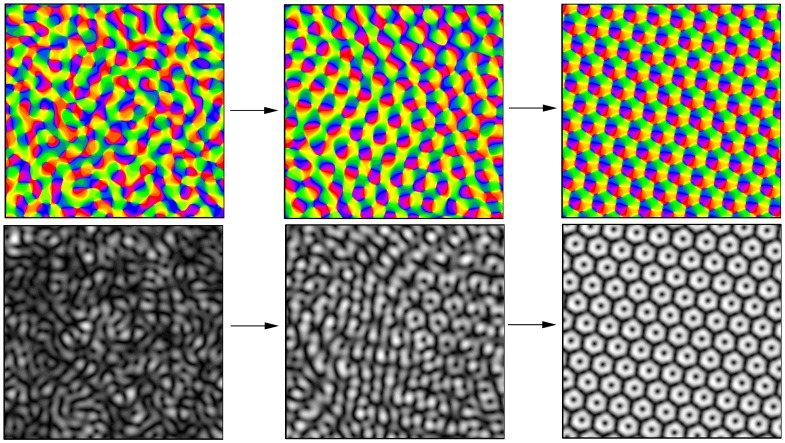
Snapshots of the pinwheel crystallization process. Top panel: OP map, bottom panel: selectivity 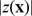. Left: 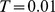, middle: 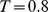, right: 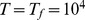. Parameters as in Fig. 4(c), 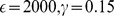.

Regions of hPWC layout can however be inter-digitated with long lived rPWC patterns and stripe domains. Figure 4**D** shows the time course of the normalized power 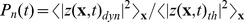, where 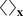 denotes spatial average. The field 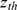 is obtained from the solution of the amplitude equations (see [39]) while 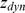 is the field obtained from the simulations. Starting from a small but nonzero power the amplitudes grew and saturated after 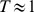. When the amplitudes were saturated the selection of the final pattern started. Quantitatively, we found that with weak backreaction the critical coupling strengths were slightly increased compared to their values in the limit 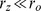. Snapshots of the simulation leading to the hPWC solutions at three time frames are shown in Fig. 5. Already at 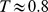 a substantial rearrangement of the pattern took place and one can identify different domains in the pattern that are locally highly stereotyped.

**Figure 6:**
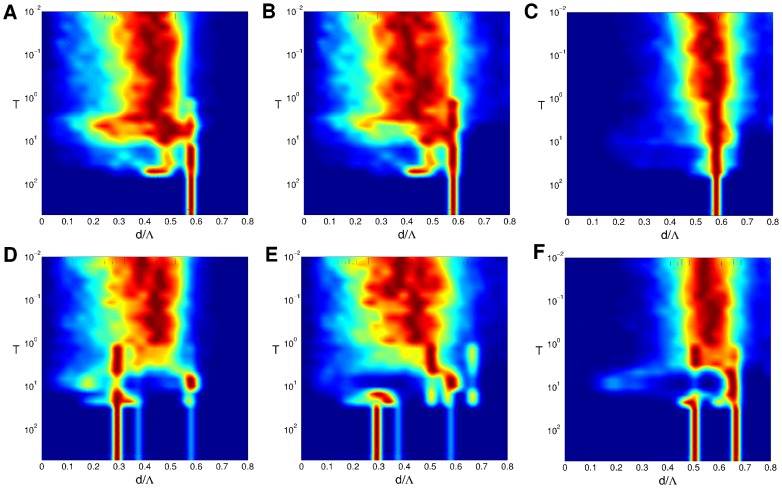
Distribution of nearest neighbor pinwheel distances during development. **A–C** rPWC **D–F** hPWC. Distance to the next pinwheel of arbitrary **A,D**, opposite **B,E**, and equal **C,F** topological charge. Parameters as in Fig. 4**B**.

For the time evolution of the maps we also calculated the distributions of pinwheel next-neighbor distances 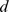, measured in units of the column spacing 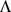. The distributions of distances for simulations leading to rhombic and hPWC solutions are shown in Fig. 6. They are characterized by three stages in the evolution of the pinwheel distances. At early stages of the evolution (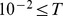) there is a continuous distribution starting approximately linearly from 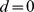. At the time where the amplitudes saturated (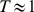) the distribution of pinwheel distances became very inhomogeneous. Different domains with stripe-like, rhombic, or hexagonal patterns appeared until for 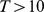 the rhombic or hexagonal pattern took over the entire system.

**Figure 7:**
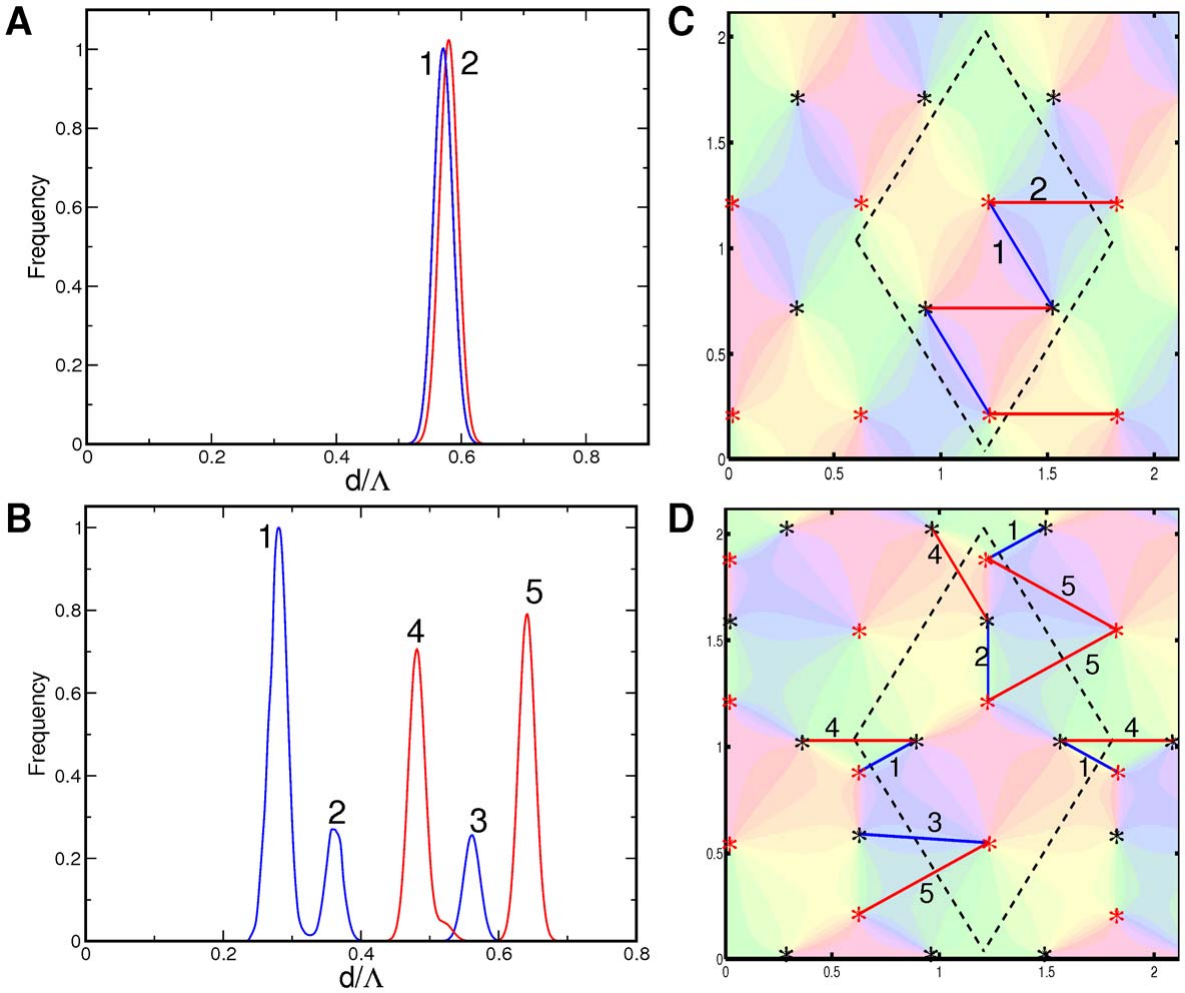
Distribution of nearest neighbor distances for final states (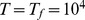). **A** rPWC, **B** hPWC with pinwheels of equal (red) and opposite (blue) charge. **C** and **D** Illustration of occurring pinwheel distances between pinwheels of equal (red lines) and opposite (blue lines) charge. Pinwheels are marked with star symbols according to their charge. Units are given in 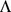. Parameters as in Fig. 4**B**.

As pinwheels carry a topological charge we could divide the distributions according to distances between pinwheels of the same charge or according to distances between pinwheels of the opposite charge. In Fig. 7 we present pinwheel distances for the final states of the dynamics. In case of the rhombic solutions there is only a single pinwheel to pinwheel distance with 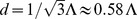. In numerical simulations small variations in the amplitudes lead to a slightly larger distance between pinwheels of equal charge than between pinwheels of opposite charge. Therefore their distance distributions do not collapse exactly, see Fig. 7**A**. In case of the hPWC there are three peaks at 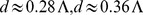 and 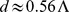 in the pinwheel distance distribution of arbitrary charge, see Fig. 7**B**. These three peaks all result from distances between pinwheels carrying the opposite charge while the distance between pinwheels of the same charge shows two peaks at 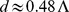 and 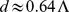 in the distribution. The origin of the peaks is indicated in Fig. 7**C** and Fig. 7**D**.

**Figure 8:**
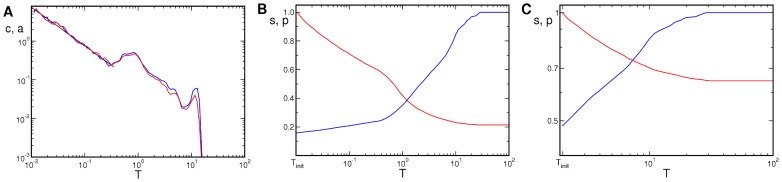
Pinwheel annihilation and creation. **A** Creation (blue) and annihilation (red) rates during time evolution. Fit: 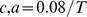 (black line). **B,C** Survival fraction (red) and fraction of preserved pinwheels (blue) compared to the initial time 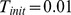 **B** and 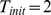 **C**. Parameters as in Fig. 4**C**.

These results confirm that inter-map coupling can induce the stabilization of pinwheels in the OP pattern. This however does not mean that the pinwheels initially generated by spontaneous symmetry breaking will be preserved during convergence of the map. To what extent are the pinwheels in the crystalline OP maps preserved from pinwheels of the initial OP pattern? To answer this question we calculated the pinwheel annihilation 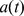 and creation 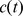 rate during time evolution, see Methods. The time evolution of these rates, averaged over 20 simulations leading to a hPWC, is shown in Fig. 8**A**. We observe that both rates were fairly similar throughout development, with a slightly higher creation rate in the later stage of development. During the initial stages of time evolution creation and annihilation rates decay algebraically 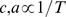. At 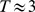 both rates deviate from this algebraic decay. From thereon annihilation and creation rates increase, reflecting the nonlinear rearrangement of the pattern. After 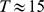 no pinwheels are created or annihilated anymore and the pinwheels of the final pattern are present.

Pinwheels are created and annihilated until a first crystal-like pattern is formed. How many pinwheels of the initial pattern are still present in the final pattern? For a given set of pinwheels at an initial time 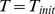 we further calculate the fraction 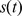 of those pinwheels surviving until time 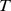. The fraction of pinwheels present at time 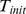 that survive up to the final time 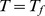 is given by 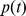. Both fractions are shown in Fig. 8**B** for 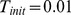 and in Fig. 8**C** for 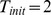, a time where the power 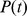 has almost saturated, see Fig. 4**D**. We observed that about 20% of the initial pinwheels are preserved until the final time and therefore most of the pinwheels of the crystal pattern are created during development. From those pinwheels which are present when the power saturates about 65% are also present in the final pattern.

### Detuning OD and OP wavelengths: OD stripes

**Figure 9:**
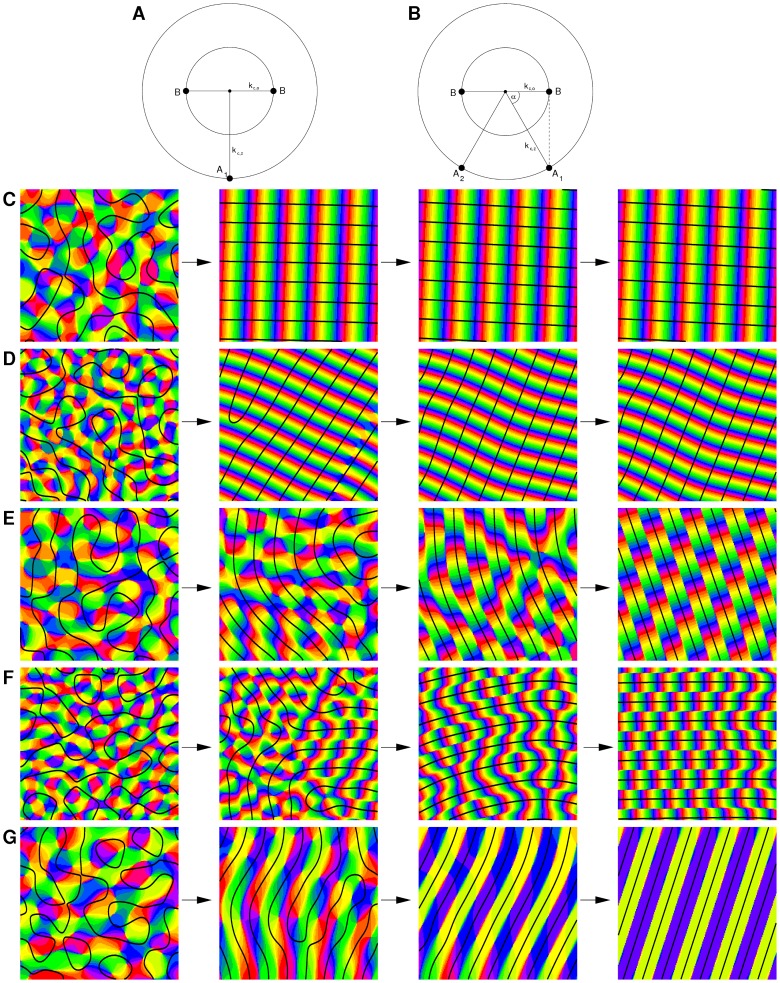
Map interactions with detuned wavelengths and OD stripes. **C–F** OD stripes interacting with OP columns where 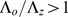. **G** OD stripes interacting with OP columns where 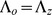. **A,B** Illustration of active modes in Fourier space with 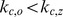, 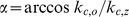. **C,D** 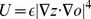, 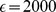, **E–G** 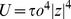, 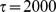, **C,E** 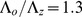, **D,F** 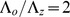. **G** From left to right: initial condition, 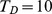, 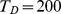, 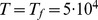. Parameters: 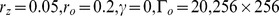 mesh.

The analytical results obtained in [39] as well as the previous numerical results (see Fig. 3**B**) predict that OD stripes do not lead to spatially complex patterns and are not capable of stabilizing pinwheels. In case of gradient-type inter-map couplings the OP map consists of stripes which run perpendicular to the OD stripes. In case of the product-type inter-map coupling high gradient regions of both maps avoid each other by producing again OP stripes but now oriented parallel to the OD stripes. In numerical simulations we also investigated the case of OD stripes of larger wavelength than OP columns, as is the case in macaque monkey primary visual cortex [7], [46]. In case of a gradient-type inter-map coupling we find that the OD bands are perpendicular to the OP bands independent of the ratio 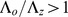, see Fig. 9**C,D** and Fig. S1. In case of the product-type inter-map coupling, if the ratio 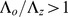, the orientation representation does not collapse as it would be the case for 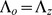, see [39]. The system, however, again finds a way to put zero contours (Re 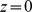 and Im 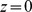) along lines of maximal OD resulting in an orientation fracture line, see Fig. 9**E,F** and Fig. S1. The angle between the active OP and OD modes is given by 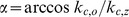 corresponding to the resonance condition 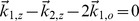, see Fig. 9**A**.

**Figure 10:**
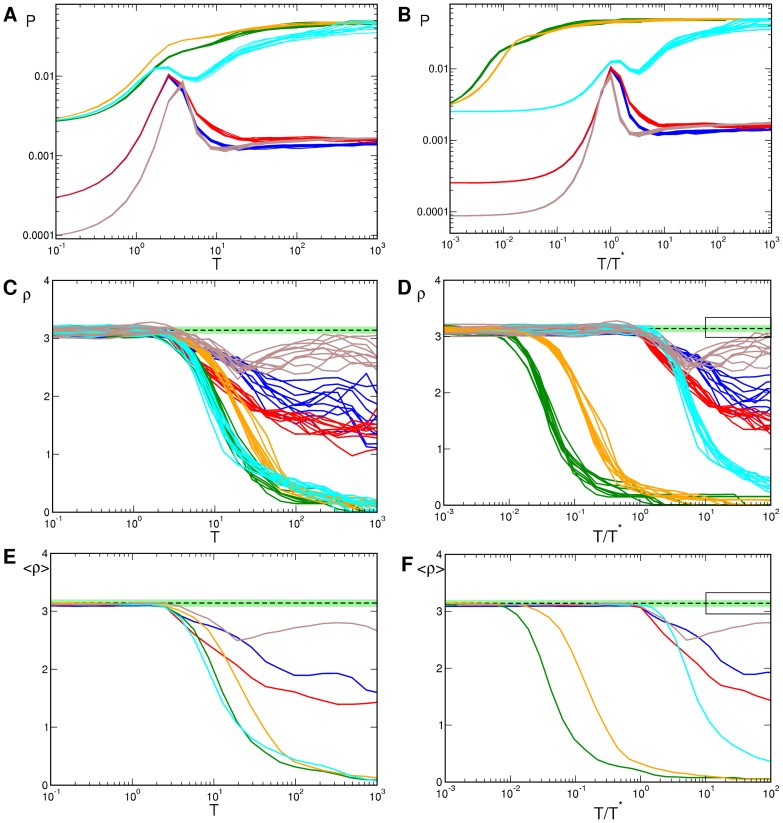
Pinwheel statistics with detuned wavelengths and OD stripes. 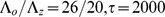
 (blue), 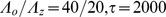 (red), 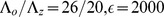 (green), 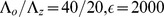 (orange), 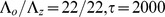 (brown), 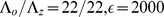 (cyan). **A,B** OP Power. **C,D** Pinwheel density. **E,F** Mean pinwheel density. Dashed line: 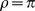. Light green region: Confidence interval of species grand average pinwheel density, see. [42]. Black rectangles indicate times 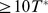. Parameters as in Fig. 9.

The time evolution of all pinwheel statistics and its comparison to the case of equal wavelengths is shown in Fig. 10 and Fig. S2. Initial conditions are band-pass filtered Gaussian white noise with initial power a few percent of the final power. Note, the pinwheel statistics are shown for the timescale 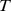 and 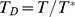 which relates the pinwheel statistics to the rise and saturation of the orientation selectivity. The power of the OP map reached about 90 percent of its final value earliest at 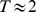. A non-monotonic time dependence of OP power can result from inter-map coupling. In particular, the rise of OD power leads to OP suppression. This suppression is absent if the pattern arranges such that the inter-map coupling energy is zero i.e. for perpendicular stripe patterns. In all cases, at 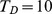 the average pinwheel density clearly deviates from the experimentally observed value. Furthermore, pinwheel densities are for all cases outside of the confidence interval of the species grand average pinwheel density obtained in [42]. Note, in case of equal wavelength and a product-type inter-map coupling energy the OP map develops towards *orientation scotoma* solutions which are selective to only two preferred orientations, see Fig. 9**G** and part (I).

### Detuning OD and OP wavelengths: OD hexagons

**Figure 11:**
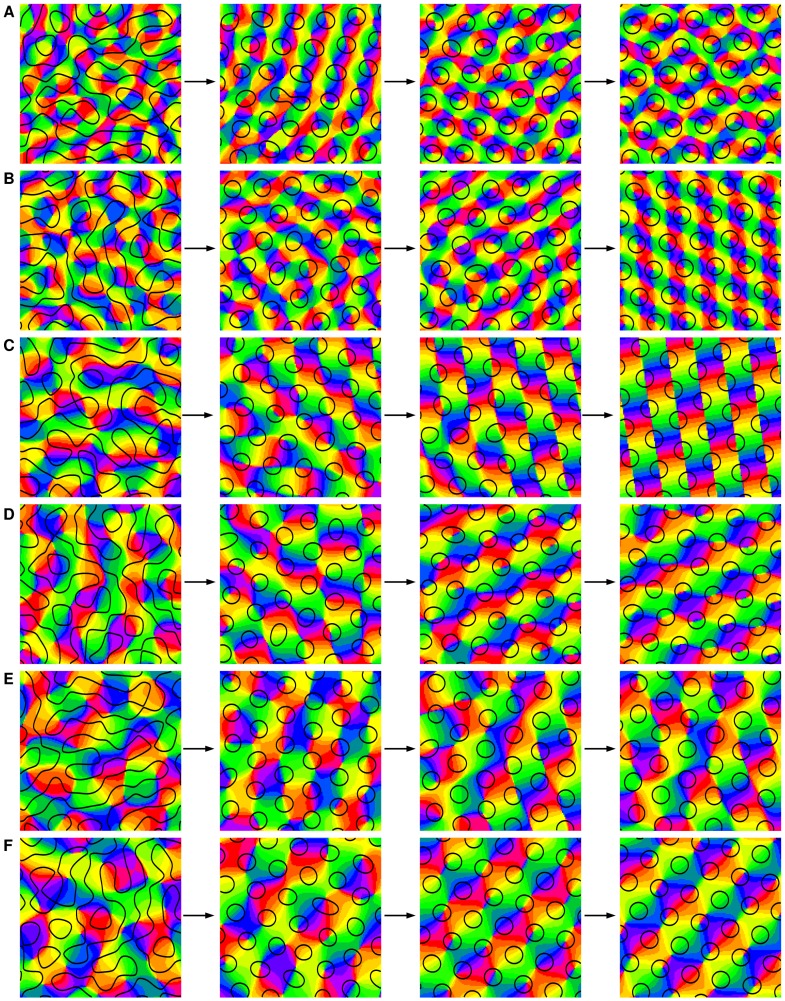
Map interactions with detuned wavelength and OD hexagons. 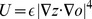
. **A** 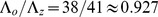, **B** 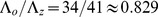, **C** 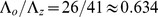, **D** 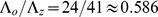, **E** 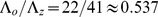, **F** 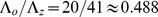. From left to right: initial condition, 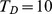, 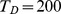, 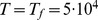. Parameters: 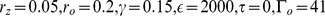, 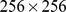 mesh. Initial condition identical in all simulations.

**Figure 12:**
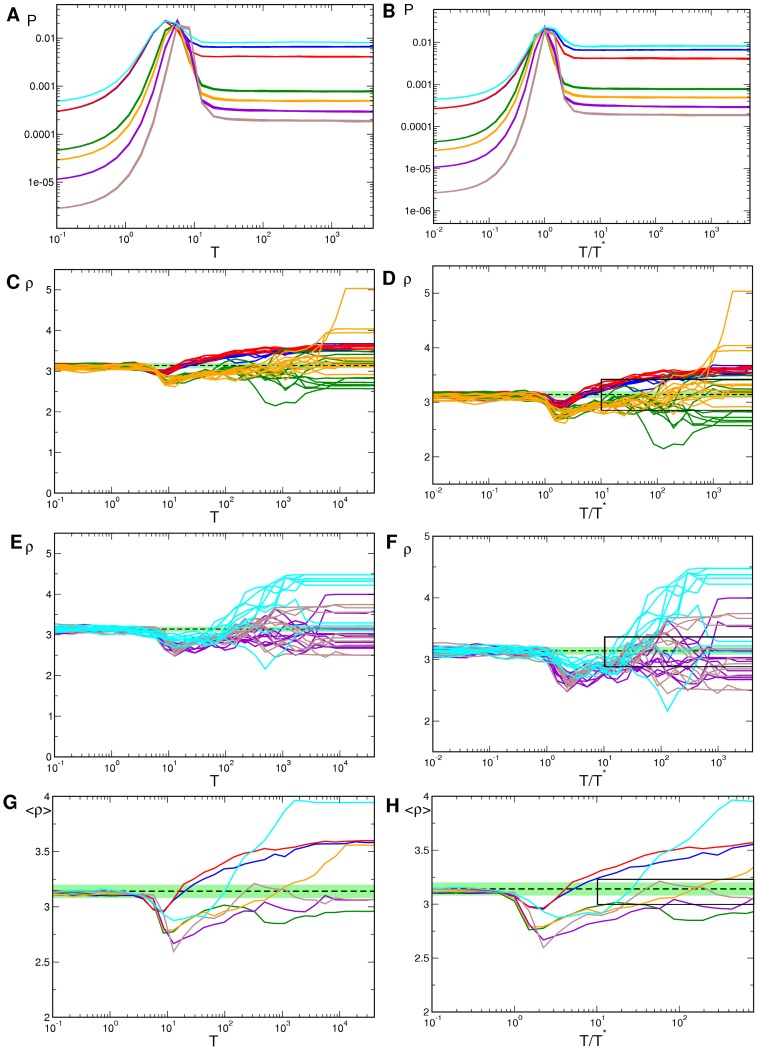
Pinwheel statistics with detuned wavelength and OD hexagons. 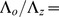
 38/41 (blue), 34/41 (red), 26/41 (green), 24/41 (orange), 22/41 (violet), 20/41 (brown), 22/22 (cyan). **A,B** OP Power. **C,D** Pinwheel density. **E,F** Mean pinwheel density. Black dashed line: 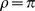. Light green region: Confidence interval of species grand average pinwheel density, see. [42]. Black rectangles indicate times 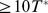. Parameters as in Fig. 11.

In case of identical wavelengths 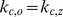 strong interaction with a system of hexagonal OD patches leads to hPWC solutions. For these solutions pinwheel positions are correlated with OD extrema. For instance in case of the higher order gradient-type inter-map coupling energy, for which the contra-center PWC corresponds to the energetic ground state, half of the pinwheels are located at OD extrema while the remaining half are located near OD borders (see Fig. S7 of part (I), [39]). If, however, the typical wavelengths of OD and OP patterns are not identical such a precise relationship cannot be fulfilled in general. We therefore studied whether a detuning of typical wavelengths can lead to spatially irregular and pinwheel rich OP patterns. In numerical simulations which lead to OD hexagons with a fixed wavelength we varied the OP wavelength using as initial conditions band-pass filtered Gaussian white noise with power a few percent of the final power. Wavelength ratios were chosen such that each pattern exhibited an integer aspect ratio. Wavelength ratios were 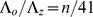 with 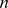 an integer. Examples of final patterns of such simulations are shown in Fig. 11 and Fig. S3 using the high order gradient-type inter-map coupling energy. In all studied cases the final patterns are spatially regular. The observed patterns are either fractured stripe patterns with two active modes Fig. 11**C** or rPWC solutions (two modes plus the corresponding opposite modes). We also studied map interactions with wavelength ratios were 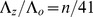 with 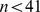. In this case, however, we found only pinwheel-free stripe patterns as final states. Much larger domains than used in the current simulations would be needed to simulate values intermediate to the wavelength ratios used here. Our results, nevertheless, clearly establish that OD induced pinwheel stabilization can occur also with detuned wavelengths. They furthermore confirm that wavelength detuning does not by itself generates irregular stable maps in the considered model. The time evolution of all pinwheel statistics and its comparison to the case of equal wavelengths is shown in Fig. 12 and Fig. S4. The pinwheel density appears to exhibit a complex dependence on the wavelength ratio. The power of the OP map reached about 90 percent earliest at 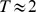. At 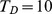, however, the average pinwheel density in all conditions clearly deviates from the experimentally observed value. Furthermore, at 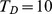 the pinwheel density for all conditions is outside of the confidence interval of the species grand average pinwheel density as obtained in [42]. For three conditions, the mean pinwheel density transiently reentered the confidence interval at a later stage for a short period of time. For no condition, however, there was a robust and stable convergence of the predicted pinwheel density to the confidence interval for 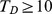. Note, in case of equal wavelength the OP map develops towards contra-center PWCs, see also Fig. 3**C,D** and part (I).

### Higher feature space dimensionality

**Figure 13:**
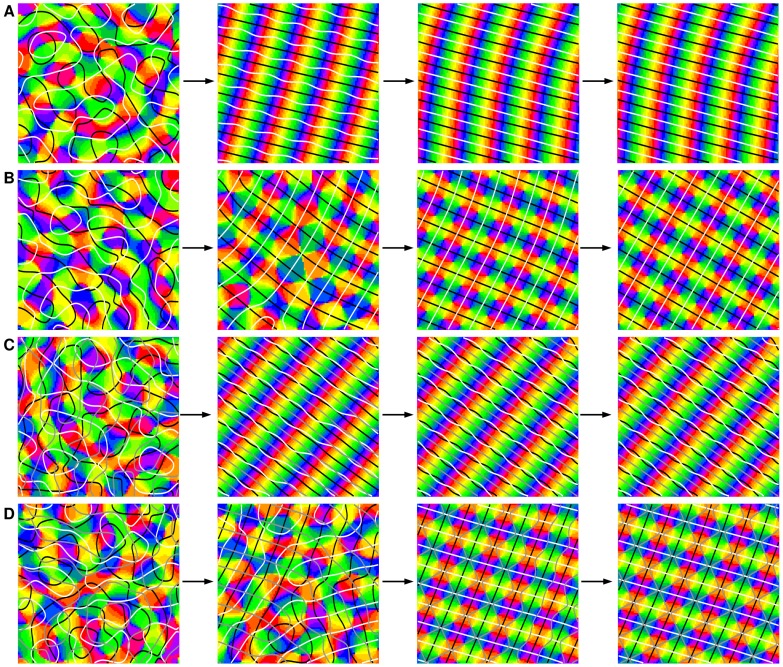
Map interactions in higher feature dimensions. **A,B** Map layout by interactions between three columnar systems (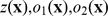). All maps are mutually coupled. Superimposed on the OP map there are the borders of two real fields (black, white). **A** 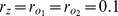 **B** 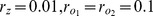. **C,D** Interactions with four columnar systems (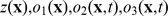). **C** 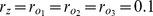. **D** 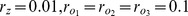. Superimposed on the OP map there are the borders the of three real fields (black, gray, white). From left to right: initial condition, 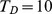, 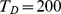, 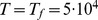. Parameters in all simulations: 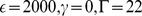, 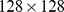 mesh.

**Figure 14:**
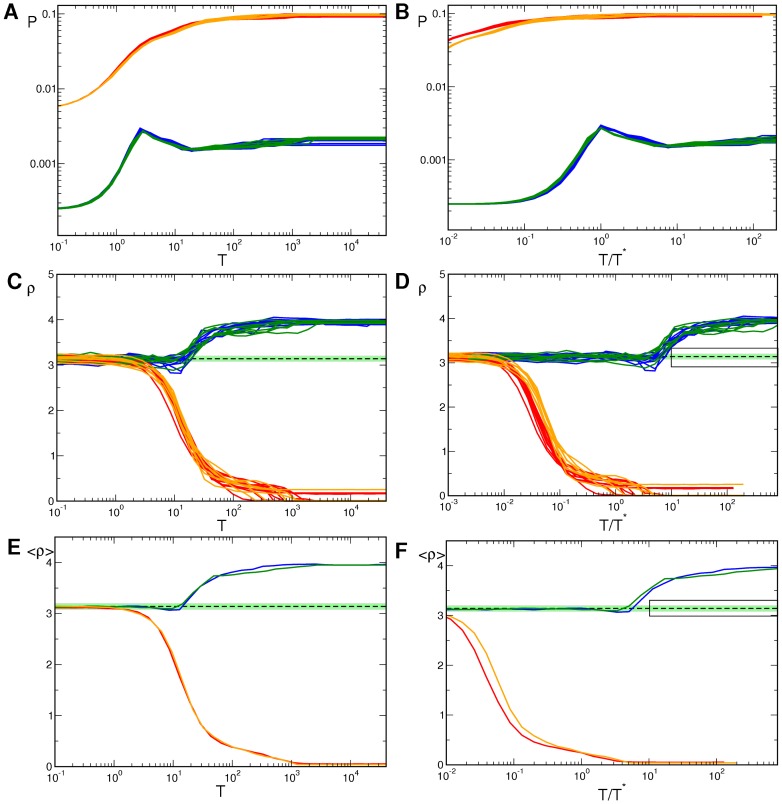
Pinwheel statistics in higher feature dimensions. Blue: 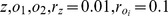, red: 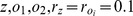, green: 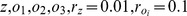, orange: 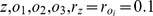. **A,B** Power of OP map. **C,D** Pinwheel density. **E,F** Mean pinwheel density. Black dashed line: 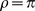. Light green region: Confidence interval of species grand average pinwheel density, see. [42]. Black rectangles indicate times 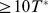. Parameters as in Fig. 13.

The inclusion of more feature dimensions into the dynamics was performed as in Eq. (9), Eq. (10) as the geometric correlations between the different types of maps seem to be qualitatively similar [9], [10], [14], [26], [40]. We used the higher order gradient-type inter-map coupling with three and four maps which are mutually coupled, see Fig. 13 and Fig. S5. Initial conditions for all maps were band-pass filtered Gaussian white noise with the initial power a few percent of the final power, see Fig. 14. Whereas in the case of two maps the coupling energy is zero if the two stripe solutions are perpendicular to each other the interactions between more maps could potentially lead to frustration as not all of the individual coupling energies can simultaneously vanish. Using the gradient coupling energy
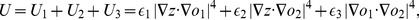
(12)no OD bias (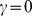), and equal coupling strengths 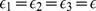 we observed two types of stationary solutions, see Fig. 13. When all bifurcation parameters were equal, the OP map consisted of stripes. Also the two real fields consisted of stripes, both perpendicular to the OP stripes i.e.
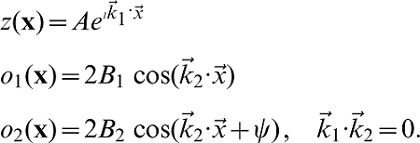
(13)The energy in this case is given by 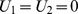, 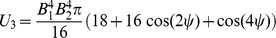 which is minimal for 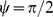, i.e. the energy is minimized by shifting one real field by one quarter of the typical wavelength. When the bifurcation parameter of the OP map was smaller than that of the two real fields we obtained PWC patterns, see Fig. 13**B**. The pinwheels were arranged such that they are in the center of a square spanned by the two orthogonal real fields and the resulting pinwheel density is 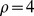. All intersection angles between iso-orientation lines and borders of the real fields were perpendicular. When extending the system by a third real field we observed a similar behavior. Figure 13**C,D** shows the stationary states of a complex field coupled to three real fields. In case of equal bifurcation parameters the stationary patterns were OP stripes, perpendicular to stripe and wavy real patterns. When the bifurcation parameter of the OP map was smaller than the other bifurcation parameters we again observed pinwheel crystallization. Note, that in this case all pinwheels were located at the border of one of the three real fields. In summary, pinwheel crystallization was only observed when the OP map is driven by the real field i.e. when the OP amplitudes are small. In all observed cases the final patterns were spatially perfectly periodic. The time evolution of all pinwheel statistics is shown in Fig. 14 and Fig. S6. The power of the OP map reached about 90 percent of its final power earliest at 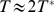. At 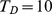 the average pinwheel density in all cases clearly deviates from the experimentally observed value. Furthermore, at 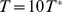 the pinwheel density in all cases is outside of the confidence interval of the species grand average pinwheel density as obtained in [42].

## Discussion

### Summary of results

In this and the accompanying analytical study, we presented a dynamical systems approach to the coordinated optimization of maps in the visual cortex such as orientation preference (OP) and ocular dominance (OD) maps. In part (I) we examined in particular the predicted optima of various candidate energy functionals [39]. We calculated phase diagrams for different energy functionals showing that for strong inter-map coupling pinwheel crystals are optima of the system. In the current study, we numerically analyzed the dynamics of two representative examples of these coordinated optimization models. We focused on the high order gradient-type inter-map coupling energy that can reproduce all qualitative relationships found experimentally between OP and OD maps, does not suffer from potential OP map suppression, and has a relatively simple phase diagram near the symmetry breaking threshold. The main phenomenon characterizing the considered models, crystallization induced by coordinated optimization and inter-map coupling, was confirmed numerically. This phenomenon was found to be robust to the influence of a weak backreaction of the OP map on the OD map, to detuning of the typical wavelengths, and was found to persist in models with higher feature space dimensionality. We characterized the complex dynamics during crystallization and calculated all pinwheel statistics known for the common design of OP maps found experimentally. The crystalline periodic layout of pinwheel-rich solutions persisted in all studied conditions. Characterizing the behavior of transients, we found that spatially irregular transient states decayed relatively fast into locally ordered patterns during optimization.

### The dynamics of developmental optimization

The optimal layouts predicted by the models considered in the current studies deviate qualitatively and quantitatively from experimentally observed map layouts. It is therefore not reasonable to assume that a genetically encoded pattern of cortical columns has been optimized on evolutionary timescales following the optimization principles formalized by our models. Two alternative scenarios, however, are raised by our results. First, visual cortical maps could be considered as optimized with respect to principles qualitatively distinct from those examined here (see e.g. [66], [67]). Second, visual cortical maps might be incompletely optimized by a developmental dynamics that reduces an energy functional such as the ones considered here but does not reach optimized states due to a finite duration of the period of juvenile plasticity. In the following we first discuss the incomplete optimization scenario and propose quantitative criteria for testing its plausibility. We then discuss likely ingredients of fundamentally different optimization principles that appear better suited to explain visual cortical architecture.

In our simulation studies, we examined the sequence of stages predicted, for the maps under the assumption of developmental optimization. Our results consistently show that our coordinated optimization models exhibit a complex dynamics that persistently reorganizes maps over different timescales before attractors or optima are reached. As can be predicted from symmetry principles [22], [68], at early stages of development maps must be spatially irregular if they develop from weakly tuned random initial conditions. Such OP patterns are essentially random exhibiting a model insensitive, universal spatial organization throughout the initial emergence of orientation selectivity. The average pinwheel density in these early maps is bounded from below by the mathematical constant 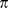 and the distributions of nearest pinwheel distances are continuous and broad. As soon as orientation selectivity started to saturate the patterns typically reorganized towards one of a few crystalline spatial patterns. This early phase of local crystallization rapidly leads to the occurrence of different spatial domains within the pattern, with a locally stereotypical periodic layout. Even in the cases exhibiting the slowest decay of irregular patterns, this process was complete after ten intrinsic timescales 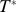. The slower dynamics that characterizes further development progressively aligns these domains leading to a long-range ordered perfectly periodic crystalline array. This long-ranged reorganization of patterns lasts substantially longer than the intrinsic timescale. Similar behavior was also observed when starting near spatially irregular unstable fixed points of the orientation map dynamics. Pinwheel crystal (PWC) solutions represent attractor states and we found no other, spatially irregular, long-living states in the dynamics. The overall progression of states observed in our models has been found previously in numerous pattern forming dynamics both highly abstract as well as in detailed ab initio simulations [59], [64], [65].

### Comparing the dynamics of coordinated optimization to stages of visual cortical development

Does the observed rapid decay of irregular OP layouts into crystalline patterns speak against the biological plausibility of an optimization dynamics of the type considered here? Can one reasonably expect that a similar crystallization process could also unfold relatively rapidly during the development of the brain? Or is it more likely that what seems rapid in our numerical simulations would take very long in a biological network - potentially so long that the cortical circuitry has already lost its potential for plastic reorganization before substantial changes have occurred? To answer these questions it is important (1) to examine whether secondary reorganization processes subsequent to the initial establishment of selectivity are occurring during biological development, (2) to delimit the fundamental timescales of the postnatal development of visual cortical circuits subserving orientation preference and ocular dominance and (3) to discuss how these timescales can be compared to the formal timescales that appear in dynamical models of map formation and optimization. In the following we address these issues. We will first summarize available evidence for ongoing pattern reorganization subsequent to the initial emergence of feature selectivity. We will then discuss the theoretically predicted properties of the fundamental time scale of the map dynamics and finally discuss how to empirically estimate it relative to the duration of visual cortical critical period plasticity. For comparing simulation results to developmental stages in the biological system the most important quantity is the relative duration of the period of juvenile plasticity; the ratio of the absolute duration of juvenile plasticity 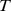 and the fundamental time scale of the map dynamics 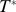. Secondary map rearrangement has been experimentally found by several studies [69]–[73]. It is expected if this ratio is substantially larger than one. How far developmental reorganization can be expected to progress towards attractor states during the period of juvenile plasticity is determined by its absolute value. Current empirical uncertainties do not permit to determine the relative duration of the period of juvenile plasticity with great accuracy. It is however, possible to estimate a conservative lower bound and a worst case estimate upper bound. We argue that plausible candidate models should correctly predict map layouts in adult visual cortex when 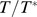 reaches the lower bound. In general model predictions should be compared to biological observations throughout the range delimited be the lower and upper bounds for a systematic assessment of the robustness of model behavior. Current data implies a conservative lower bound to the duration of the period of juvenile plasticity of about 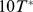.

### Juvenile plasticity supports an ongoing reorganization of OP and OD

Accumulating evidence suggests that juvenile plasticity supports an ongoing pattern reorganization [69]–[73]. For cat visual cortex, Kaschube and coworkers have demonstrated that the spatial organization of orientation columns in striate cortex is progressively reorganized between the sixth and the 14th postnatal week such that the organization of orientation columns that are reciprocally connected to extra-striate visual cortex and contra-lateral hemisphere striate cortex are better matched [70]. A second line of evidence is related to the fact that the surface area of cat striate cortex substantially increases postnatally [71], [74]–[76]. The spatial periodicity of both orientation as well as OD columns, however, remains basically unaffected during this period [70], [71], [77]. Keil and coworkers reported that this areal growth in the presence of maintained mean column spacing induces a specitifc kind of spatial reorganization of the layout of OD columns within cat striate cortex [71]. Independently, growth related rearrangement of orientation columns has also been suggested previously by Kiorpes and coworkers from observations on a smaller data set from juvenile macaques [46].

Perhaps the most striking demonstration that the functional preferences of visual cortical neurons can reorganize over long time scales during the period of juvenile plasticity has emerged from studies of the mouse visual cortex. In the mouse, as in cat, visual cortical neurons first develop orientation selectivity around the time of first eye opening in the second postnatal week [69], [72], [73]. Similar to the developmental time course in the cat, the duration of the period of juvenile plasticity in the mouse is quite long and extends beyond the third postnatal month [78], [79]. At a duration of more than 10 weeks, it is thus substantially longer than required for the expression of adult-like single neuron selectivities. Wang and coworkers demonstrated that neurons in the binocular segment of mouse visual cortex change their preferred orientations during this period [69]. Neurons in the binocular segment of mouse striate cortex were found to first exhibit widely different preferred orientations in the left and right eye. The two different preferred orientations then underwent secondary reorganization and became matching at an age of 5 weeks postnatally after the peak of the OD critical period [69]. In the monocular segment of mouse visual cortex, Rochefort and coworkers found substantial changes in the complement of preferred orientations and preferred directions represented during the first postnatal month [72]. It is noteworthy that a substantial long-term reorganization of cortical preferences has also been demonstrated for the preferred direction for whisker deflection in the rat barrel cortex [80]. Here Kremer and coworkers found that preferences for the direction of whisker deflection reorganize over the course of the first three postnatal months. Long-term reorganization might thus potentially constitute a general feature of sensory cortical representations.

### Timescales of developmental plasticity

Experimental evidence thus clearly supports that circuits in sensory cortical areas remain in a state of flux for weeks and months after the initial emergence of sensory responsiveness and stimulus selectivity. To judge how far the rearrangement of cortical circuitry can progress towards a stationary optimized state one has to relate the duration of the period of juvenile plasticity to the fundamental timescale of the map dynamics. This time scale essentially is the duration of the process of establishing mature levels of response selectivity and in our models is the time 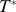. Let us first discuss the determinants of this time scale from a theoretical perspective. All models for the development of visual cortical functional selectivity from an unselective or weakly selective initial condition are known to exhibit a distinct intrinsic time scale for the emergence of stimulus selectivity in individual neurons, see e.g. [36], [50], [52], [81]–[83]. In the abstract order parameter models used here this time scale is set by the inverse of the maximum eigenvalue 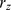. It is important to note that this time scale represents an effective parameter describing a collective circuit property. Consequently this parameter is not rigidly related to any particular cellular or synaptic time constant such as e.g. the characteristic times required for the expression of LTP, spine growth, homeostatic plasticity or other functional or morphological synaptic changes. Theoretical studies of microscopic models in which the effective time scale was explicitly calculated established that the intrinsic time scale depends (1) on the ensemble of activity patterns driving development and also (2) on characteristics of the local cortical circuits [57], [83], [84]. For instance in a representative, analytically solvable microscopic model for the emergence of OD patterns the maximum eigenvalue is given by
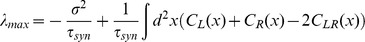
where 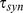 is the characteristic time scale for synaptic changes, 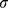 the spatial extend of co-activated neuron groups in the model cortex and 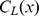, 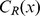 and 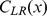 are auto- and cross-correlation functions of the activity patterns in the left and right eye layers of the LGN [57]. The effective time scale for the emergence and saturation of response selectivity is thus expected not to be faster than the fundamental processes of synaptic change. A broad range of time constants, however, is in principle consistent with Hebbian models of sensory cortical development depending on details of circuit interactions.

### A lower bound to the relative duration of juvenile plasticity

An empirical estimate for the relative duration of juvenile plasticity can be obtained by comparing the characteristic time scale of initial map emergence and the duration of the period from map emergence to the closure of critical periods for visual cortical plasticity. How long does the emergence of stimulus selectivity in the visual cortex take under normal conditions? For orientation selectivity in the primary visual cortex this information has been experimentally obtained for cat and ferret. In both species orientation selectivity is established starting from an initial condition in which cells are only weakly orientation biased. Data indicate a period between a few days and at most one week to reach mature levels of single cell orientation selectivity [11], [17], [21], [85]–[88]. Similar time scales are sufficient for substantial morphological changes of thalamo-cortical axonal structure [89]. A conservative estimate for the intrinsic time scale of map dynamics is thus that 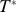 is about one week.

As mentioned above the period of juvenile cortical plasticity is known to last substantially longer. Best established experimentally is the period of susceptibility to monocular deprivation in the cat that lasts for several months of postnatal life [86], [90]–[95]. The primary visual cortex of the cat is maximally susceptible to monocular deprivation in kitten of four weeks of age [90]–[92]. An initial establishment of OP and OD maps in kittens occurs around the time of eye opening and is complete at the end of the second postnatal week [85], [87]. A maximal degree of plasticity is thus reached two weeks after the emergence of maps and the onset of natural vision. After the first postnatal month susceptibility to monocular deprivation gradually declines back to levels comparable to those present at the onset of vision and initial map emergence. The closure of the period of developmental plasticity was estimated by three independent studies to occur between the 14th and 18th postnatal week [90]–[92]. This is 12 to 16 weeks after single neurons first exhibit adult like levels of orientation selectivity and eye dominance. The reported durations for the period of juvenile plasticity thus are 12 to 16 fold of our conservative estimate for the fundamental time scale. A lower bound for the relative duration of juvenile plasticity is thus 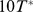. According to this estimate, plausible candidate models should thus predict map layout consistent with biological observations in the adult at 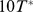.

### The fundamental timescale is likely to speed up towards the peak of the critical period

The simulations presented in the current studies were designed to assess the convergence of model maps to final attractor states. We presented our results in a way that enables comparison of predicted and biologically observed layouts throughout a broad time span including 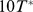 but also extending beyond this stage. This approach enables to assess the dynamical stability and robustness of the layout obtained.

Considering this robustness is not only interesting for theoretical reasons. Experimentally it cannot be excluded that the relative duration of the period of juvenile plasticity is substantially longer that the lower bound estimated above. The fact that the maximal level of plasticity is observed not at eye opening but two weeks later means that similar size changes will unfold on shorter time scales as the peak of the critical period is approached. Several studies have attempted to assess the fundamental time scale for the establishment of stimulus selectivity near the peak of the critical period. Classical studies by Blakemore and Mitchell [96], and Imbert and coworkers [97]–[99] examined how much visual experience is needed to achieve selectivity from an unselective initial condition at the time of peak OD plasticity. To this end they examined the newly generated selectivity of neurons in kitten dark-reared until the peak of the OD critical period and then given short epochs of normal visual experience. These studies indicate that 6 hours of visual experience are sufficient to induce a substantial degree of selectivity in visual cortical neurons. Recent studies provide further evidence for relevant time scales of visual cortical plasticity on such an accelerated time scale. Mitchell, Sengpiel and coworkers examined how many hours of normal visual experience are sufficient to prevent gross neuronal changes of selectivity and impairment of perceptual abilities under visual deprivation [100]–[103]. They report that two hours of normal visual experience per day are sufficient to completely prevent deprivation induced impairments of visual function. Directly imaging the emergence of direction preference columns in a network initially lacking such columns has been achieved by Li et al. in juvenile ferrets [104]. This study found that even under anesthesia, balanced visual stimulation over 3–6 hours was sufficient to drive the *de novo* formation of a system of columns. These novel experiments fundamentally differ from previous pairing studies [105]–[108] in that visual cortical neurons were not artificially trained to adopt a particular stimulus preference but were stimulated by a set of opposing motion patterns. Neurons were not artificially activated and were free to develop preference and anti-preference for any stimulus from a balanced set. It appears unlikely that this stimulation paradigm artificially accelerates changes. A corresponding process in the brain of an awake and attending animal is thus expected to be orders of magnitude slower. These studies thus substantially extend prior observations in anesthetized animals in which visual cortical neurons were artificially activated. Also under these more artificial conditions visual cortical preferences for stimulus orientation, direction, or preference for one eye were found to undergo substantial activity induced changes within a few hours [105]–[108].

Various studies thus confirm the basic expectation that around the peak of the critical period the typical time scale for the emergence of selectivity from unselective network states and for changes of selectivity is substantially shorter than one week. In particular, in light of these results it is required to assess the robustness and dynamical stability of model predictions beyond 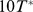. A pessimistic estimate for the resulting prolongation of realistic simulations can be obtained by assuming that the accelerated time scale is effectively relevant for the most of the 10–14 week period of juvenile plasticity. Assuming 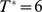 h, larger that the times reported in the above experiments, the duration of the period of juvenile plasticity would correspond to 280–370 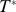.

### An absolute upper bound for the relative duration of juvenile plasticity

The above considerations are a useful reminder that that the current understanding and experimental characterization of the timescales of circuit dynamics are substantially limited. New experimental approaches that provide a more direct and certain assessment of what one might call circuit turnover times, would in fact be very informative for calibrating dynamical and optimization studies. In the absence of such information, it appears also useful to estimate a maximal upper bound to the relative duration of juvenile plasticity that is very unlikely to be ever overturned by future improvements in experimental technology. Such a worst-case estimate is obtained by the ratio of the longest duration ever reported for critical period plasticity in the visual cortex to the low end of the accelerated time scales. To our best knowledge the longest estimate for a critical period in cat visual cortex was obtained by Jones, Spear and Tong [93]. These authors examined juvenile cats deprived at older ages than in the classical studies cited above in an attempt to determine whether visual areas higher in the cortical processing hierarchy exhibit a delayed or extended period of developmental plasticity. They reported that substantial modifications of responses could still be induced up to the 35th postnatal week [93]. Remarkably their results indicate that the period of susceptibility extends longer in the primary visual cortex than in areas higher in the visual processing hierarchy. This would amount to an entire duration of the period of juvenile plasticity of 33 weeks. Assuming a fundamental timescale in the lower range of the experimentally reported peak critical period time scales i.e. 3h one estimates an absolute upper bound for the relative duration of the period of juvenile plasticity in the cat of 1850 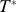.

### When does a dynamical model successfully explain adult functional cortical architecture?

Even in light of the most conservative considerations presented above it appears of limited value to compare maps from a simulation obtained when selectivity first reaches mature levels to biological patterns present in the adult cortex. Maps in the adult visual cortex of the cat have been subject to more than ten weeks of ongoing plasticity. They are thus better viewed as dynamic equilibria that are largely maintained under a continuous process of ongoing activity-driven synaptic turnover. Current experimental evidence, nevertheless, indicates that the maps emerging initially over the first days of normal vision exhibit many layout properties that are preserved throughout the juvenile period of plasticity and into adulthood [11], [42]. Taking the long duration of the period of juvenile plasticity into account, this is likely to mean that these properties have been actively maintained by an ongoing dynamics. The requirement to generate and maintain a realistic column layout is a more selective criterion for the identification of appropriate candidate models than the mere ability to initially generate good looking maps as demonstrated by our current results as well as many prior theoretical studies (reviewed in [42], supplement). It is thus a more stringent test of a models explanatory power to compare the maps obtained at later stages, e.g. 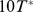 to the biologically observed functional organization of the visual cortex. Our estimates suggest that it is reasonable to require of a biologically plausible model that states which resemble the adult functional architecture are predicted at least one order of magnitude later than the maturation of average selectivity. Using this criterion, the states observed in our simulations in fact suggest that the considered models are not capable of explaining the biological organization in a satisfying fashion: Patterns observed after 10 intrinsic timescales are dominated by crystalline local arrangements that are distinctly different from the spatially irregular layout of orientation maps observed in both juvenile and adult visual cortex. Quantitatively layout parameters at this time substantially deviate from biological observations.

### Pinwheel stabilization by map interactions

The numerical studies presented here further elucidate the conditions for pinwheel stabilization by map interactions. The analytical results presented in part (I) showed that in several models OD stripes are not able to stabilize pinwheels near symmetry breaking threshold and for only one real-valued scalar field. This result appeared to be insensitive of the specific type of inter-map interaction [39]. Our numerical results show that this result is also insensitive to a detuning of typical wavelengths. For different ratios of the typical wavelengths of OP and OD, pinwheel-rich patterns either decay into pinwheel-free OP stripes or patterns with OP fracture lines when interacting with OD stripes. These findings support the conclusion that in models for the joint optimization of OP and OD maps a patchy OD layout is important for pinwheel stabilization by crystallization.

In the current study we also generalized our dynamical systems approach to include any additional number of columnar systems. One reason to consider additional visual cortical maps originates from the finding that the removal of the OD map in experiments does not completely destabilize pinwheels [29]. Moreover, in tree shrews, animals which completely lack OD columns, OP maps contain pinwheels and exhibit a pinwheel arrangement essentially indistinguishable from species with columnar OD segregation [13]. This might reflect the influence of additional columnar systems such as spatial frequency columns that can be expected to interact with the OP map in a similar fashion as OD columns [40]. From a theoretical perspective, one might suspect that couplings between more than two systems that promote a mutually orthogonal arrangement are harder to satisfy the more maps are considered. In principle this could lead to the emergence of irregular patterns by frustration. In numerical simulations we examined coordinated optimization with three and four columnar systems. In these cases pinwheel stabilization is possible even without an OD bias. The resulting stationary OP patterns are, however, still either stripes or PWC solutions. For more than two feature maps, asymmetry of one feature dimension is thus not a necessary condition for pinwheel stabilization by coordinated optimization.

We also characterized the dynamics of pinwheel crystallization from pinwheel-free initial conditions. With the analytical approach presented in part (I) we were able to show that pinwheel-rich solutions correspond to the energetic ground state of our models for large inter-map coupling [39]. This can be confirmed by simulations in which pinwheels are created even when starting from an initial OP stripe pattern. Assessing pinwheel creation from pinwheel-free initial conditions could more generally serve as a simple test for the existence of a pinwheel-rich attractor state in models of OP development that can be applied to models of arbitrary complexity. One should note, however, that the production of pinwheels from a pinwheel-free initial condition provides only a sufficient but not a necessary criterion to verify the existence of a pinwheel-rich attractor state. This criterion may be violated if pinwheel-free and pinwheel-rich attractor states coexist. Nevertheless, the pinwheel production criterion can be used to demonstrate that pinwheels are not just a remnant of random initial conditions.

### Pinwheel crystallization in other models

Pinwheel crystals have been previously found in several abstract [109]–[111] as well as in detailed synaptic plasticity based models [81], [112], [113]. Remarkably, in a model of receptive field development based on a detailed dynamics of synaptic connections the resulting OP map showed a striking similarity to the hPWC presented above, compare Fig. (7) and [39], [112]. These observations indicate that pinwheel crystallization is not an artifact of the highly idealized mathematical approach used here. In fact, the first OP map predicted ever by a synaptically based self-organization model presented by von der Malsburg in 1973 exhibited a clearly hexagonal column arrangement [81]. Von der Malsburgs calculations as well as those presented in [112] utilized a hexagonal grid of cells that may specifically support the formation of hexagonal patterns. Our numerical and analytical results clearly demonstrate that patterns of hexagonal symmetry do not critically depend on the use of a hexagonal grid of cells as our simulations can generate hexagonal patterns also for square lattices of cells. To determine whether the hexagonal layout in Von der Malsburgs model is intrinsically stable it should be implemented for other grids both of square symmetry as well as for irregular positions of cells. In our study, we examined whether the non-crystalline layout of visual cortical maps could result from a detuning of OP and OD wavelengths. However, while destabilizing hPWC solutions, wavelength detuning leads to spatially regular solutions in all studied cases. This suggests that a spatially regular layout is not an exceptional behavior in models for the coordinated optimization of visual cortical maps that would require fine tuning of parameters.

### Is there evidence for hexagonal orientation column patterns?

Recently, Paik and Ringach have argued that a roughly hexagonal arrangement of iso-orientation domains would provide evidence for a defining role of retinal ganglion cell mosaics for the spatial arrangement of orientation columns [114]–[116]. It is interesting to consider this claim in view of our results as well as in view of the wealth of activity-dependent models that predict hexagonal arrangements irrespective of the arrangement of retinal ganglion cells [66], [67], [81], [112], [117]–[119]. Since all of these distinct models are known to generate hexagonal arrays of orientation columns it seems questionable to view evidence for a hexagonal arrangement as evidence for a particular activity-independent mechanism. Our characterization of the dynamics of crystallization, however, enables to identify more selective predictions of a retinal ganglion cell mosaic based formation of hexagonal iso-orientation domains. If the pattern of OP columns was seeded by retinal ganglion cell mosaics, as initially proposed by Soodak [120] and recently re-articulated by Paik and Ringach [114], hexagonal structures should be detectable from the very beginning of development, i.e. already at stages when orientation selectivity is still increasing. Hexagonal PWCs in self-organizing models, in contrast, form from an initially irregular and isotropic state. Thus the time dependence of hexagonal-like column arrangements can distinguish in principle between self-organized as opposed to retinal ganglion cell mosaic imprinted hexagonal arrangements. As we found in all models examined hexagonal arrangements are frequently of rather high pinwheel density of about 5 pinwheels per hypercolumn. Also the hexagonal pattern constructed by Paik and Ringach appear to exhibit relatively high pinwheel density of 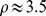. Thus both theories appear inconsistent with observed pinwheel densities. A mixed scenario in which retinal ganglion cell mosaics seed the initial pattern of iso-orientation domains and later activity-dependent refinement drives a rearrangement of OP maps towards the experimentally observed design therefore predicts a substantial degree of net pinwheel annihilation. Kaschube et al. presented evidence for essentially age independent pinwheel densities in ferret visual cortex between week 5 and 20. No indication of substantial pinwheel annihilation is visible in this data [42]. One should note that in ferret visual cortex orientation columns first arise in the fifth postnatal week [11]. The relation of the analysis by Paik and Ringach and the statistical laws described by Kaschube and coworkers ask for further analysis and comparison.

### Stabilization of spatially irregular layouts

The reason for the substantial differences to experimentally observed maps might thus be the presence of biological factors neglected in the models examined here. Candidate factors are a greater distance from the pattern formation threshold, different kinds of biological noise, or the presence of long-range neuronal interactions. Vinals and coworkers demonstrated for the case of stripe patterns that a Swift-Hohenberg model sufficiently far from the bifurcation point can exhibit stable disclination defects [121]. Although the results indicate only a spatially sparse set of stabilized defects it will be interesting to examine whether this also applies to the case of multidimensional coupled models and to establish which properties the model solutions develop very far from the bifurcation point. Theoretically, it is well understood that in principle so called ‘nonadiabatic effects’ can induce the pinning of grain boundaries in pattern forming media [122]–[125]. In one spatial dimension and for models with several interacting order parameter fields similar mechanisms may even lead to the emergence of spatially chaotic solutions [126]–[128]. These studies suggest to examine whether spatial incommensurability far from threshold can induce spatially chaotic patterns in one and two dimensional coordinated optimization models. Such studies may uncover a completely novel scenario for explaining the emergence of spatially irregular states in models of cortical map optimization.

A second interesting direction will be the inclusion of frozen spatial disorder in models for the self-organization of multiple cortical maps. Such disorder could represent a temporally fixed selectivity bias that favors particular feature combinations at different position in the cortical sheet. For OP, the proposals of Waessle and Soodak recently revisited by Ringach and coworkers that retinal ganglion mosaics might constrain and seed orientation column patterns would represent a specific mechanisms for such a fixed local bias [114]–[116], [120], [129]. Experimental evidence that retinal organization can impose local biases was revealed by Adams and Horton's finding that the pattern of retinal blood vessels can specifically determine the layout of OD columns in squirrel monkey visual cortex ([130], [131], for a modeling study see also [132]).

Spatial disorder terms in dynamical models can also be designed to represent randomness in the interactions between neurons at different positions. This might result from heterogeneities in lateral interactions within the cortical sheet. In particular this later type of modification has been examined in simple examples of order parameter equations and was found to qualitatively change the type of the bifurcation and the nature of the unstable modes [133]–[136]. It will be important to investigate how different types of spatial disorder modify the behavior of models derived from biologically meaningful energy functionals. We hope that for such studies of the influence of ‘biological noise’ a thorough understanding of the properties of perfectly homogeneous and isotropic systems as achieved here will provide a solid basis for disentangling the specific contributions of randomness and self-organization.

Finally, a third promising direction for modifying the type of models considered here is the inclusion of long-ranging intra-cortical interactions in the equations for the individual order parameter fields. The impact of long-ranging intra-cortical interactions has been studied previously both with respect to the properties of patterns emerging during the phase of initial symmetry breaking [32], [137]–[139] as well as for its influence on long-term pinwheel stabilization and pattern selection [66], [67], [140]. Models for orientation maps that include orientation-selective long-ranging interactions exhibit a good quantitative agreement of both attractors and transient states to the biological organization of OP maps in the visual cortex [42]. Including orientation-selective long-ranging interactions in models for the coordinated optimization of multiple cortical maps could provide a transparent route towards constructing improved models for the coordinated optimization of column layouts matching the spatial structure of orientation maps.

### Modeling areal borders and experimentally induced heterogeneities

Viewed from a practical perspective, the presented theoretical approach offers also convenient ways to model the impact of spatial inhomogeneities in the visual cortex on OP map structure. For this purpose, the co-evolving field does not represent a feature map but would be designed to describe a real or artificial areal border or a disruption of local circuitry. To this end, the OP map would be coupled, using low order coupling energies, to a fixed field describing the areal border such that its values are for instance one inside and minus one outside of the area with a steep gradient interpolating between the two. Outside the areal border a strong coupling to such a field can lead to complete suppression of orientation selectivity. Using a gradient-type inter-map coupling energy inter-map coupling can also be used to favor a perpendicular intersection of iso-orientation lines with the areal borders as observed in some experiments [13], [141]. Artificial heterogeneities and areal borders have been induced by local ablation or other local interventions [141], [142]. Viral approaches such as the silencing of cortical regions by transfection with hyperpolarizing ion-channels now make it possible to impose such heterogeneities with minimal intervention and potentially in a reversible fashion [143], [144].

### A hierarchy of visual cortical maps

In the current studies we focused on a particular hierarchy of visual cortical maps. In the analytical calculations and most simulations the OD map was assumed to be dominant which corresponds to a choice of control parameters that satisfy 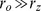. That maps form a hierarchy under such conditions can be seen from the limiting case in which inter-map interactions become effectively unidirectional. In this case the dynamics of the OP map is influenced by OD segregation while the OD dynamics is effectively autonomous. This limit substantially simplifies the analysis of map-interactions and the identification of ground states. The effect of a backreaction on the OD map can be studied within the presented approach either by solving amplitude equations numerically or by solving the full field dynamics. We observed that, although the presented optima persist, with increasing backreaction on the OD map the minimum inter-map coupling strength necessary for the stability of hexagonal pinwheel crystals increases. By solving the full field dynamics numerically we confirmed this conclusion. In the presented numerical simulations the backreaction, however, was relatively small. The simulations nevertheless establish that our results are not restricted to the limit 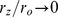 i.e. that limit is not singular. A comprehensive analysis of the effect of strong backreaction is beyond the scope of the current study. One should note that the decoupling limit 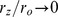 does not lead to completely unrealistic OD patterns. In particular, compared to the architecture of macaque visual cortex the uncoupled OD dynamics has stationary patterns which qualitatively resemble the layout of observed OD maps. Macaque primary visual cortex appears to exhibit essentially three different kinds of OD patterns: Fairly regular arrays of OD stripes in most of the binocular part of the visual field representation, a pattern of ipsilateral eye patches in a contralateral background near the transition zone to the monocular segment and of course a monocular representation in the far periphery. These qualitatively correspond to the three fundamental solutions of the OD equation: stripes, hexagons, and a constant solution, which are stable depending on the OD bias (see Fig. 16 of [39]). In cat visual cortex the observed OD layout is patchy throughout V1 [49], [145]–[148].

### Conclusions

The presented models for the coordinated optimization of maps in the visual cortex, that were studied analytically in part (I) and numerically in part (II), lead in all studied conditions to spatially regular energy minima. In local regions on the order of a few hypercolumn areas, column layout rapidly converges to one of a few types of regular repetitive layouts. Because of this behavior the considered models cannot robustly explain the experimentally observed spatially irregular common design of OP maps in the visual cortex. As expected from these qualitative differences all pinwheel statistics considered, exhibit substantial quantitative deviations from the experimentally observed values. These findings appear robust with respect to finite backreaction, detuning of characteristic wavelengths, and the addition of further feature space dimensions. Recent work demonstrated that the spatially irregular pinwheel-rich layout of pinwheels and orientation columns in the visual cortex can be reproduced quantitatively by models that represent only the orientation map but include long-range interactions [42], [66], [67]. In the visual cortex of species widely separated in mammalian evolution we previously found virtually indistinguishable layout rules of orientation columns that are quantitatively fulfilled with a precision of a few percent [42]. In view of these findings the current results suggest that models in which pinwheel stabilization is achieved solely by coordinated optimization and strong inter-map coupling are not promising candidates for explaining visual cortical architecture. In order to achieve a quantitatively more viable coordinated optimization theory one might consider taking additional ‘random’ factors into account. One should however not conclude that coordinated optimization does not shape visual cortical architecture. Using the general approach developed here it is possible to construct a complementary type of models in which the complex OP map is dominant. Such models, using non-local terms in the energy functional of the OP map, can be constructed to reduce to the model in [42], [66], [67] in the weak coupling limit. Because long-range interaction dominated models can reproduce the spatially irregular layout of OP maps, one expects from such models a better reproduction of the observed architecture for weak coupling. Alternative scenarios might emerge from the inclusion of quenched disorder or very far from the pattern formation threshold. Because of its mathematical transparency and tractability the approach developed in the present studies will provide powerful tools for examining to which extend such models are robust against coupling to other cortical maps and to disentangle the specific contribution of coordinated optimization to visual cortical architecture.

## Methods

### Tracking and counting pinwheels

During the evolution of OD and OP maps we monitored the states from the initial time 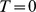 to the final time 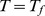 using about 150 time frames. To account for the various temporal scales the dynamics encounters the time frames were separated by exponentially increasing time intervals. Pinwheel centers were identified as the crossing of the zero contour lines of the real and imaginary parts of 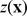. During time evolution we tracked all the pinwheel positions and, as the pinwheels carry a topological charge, we divided the pinwheels according to their charge. The pinwheel density 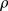 is defined as the number of pinwheels per unit area 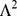. By this definition, the pinhweel density is independent of the spacing of columns and dimension-less. The distribution of pinwheel distances indicates the regularity and periodicity of the maps. Therefore we calculated the minimal distance between pinwheels, measured in units of the column spacing 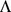, during time evolution. In simulations we used periodic boundary conditions. In order to correctly treat pinwheels close to map borders we periodically continued the maps. Nearest neighbors of pinwheels are thus searched also in the corresponding periodically continued maps.

To calculate pinwheel density variability in subregions of size 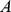 we sampled for each map circular shaped regions of various size and placed their centers at random locations of the map. Sizes of circular regions were uniformly distributed. To calculate pinwheel density variability for a given area 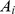, we randomly selected from all regions in the set up to 1000 regions with size in the interval 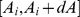 where 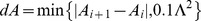, and calculated the standard deviation SD of pinwheel densities. To characterize density variability as a function of area size we estimated the variability coefficient 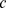 and the exponent 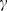 by fitting the function 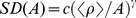 to the SD(A)-curves.

The rearrangement of OP maps leads to annihilation and creation of pinwheels in pairs. Between two time frames at 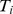 and 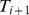 we identified corresponding pinwheels if their positions differed by less than 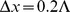 and carry the same topological charge. If no corresponding pinwheel was found within 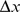 it was considered as annihilated. If a pinwheel at 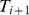 could not be assigned to one at 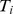 it was considered as created. We define the pinwheel creation 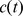 and annihilation 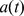 rates per hypercolumn as
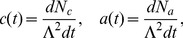
(14)where 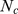 and 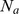 are the numbers of created and annihilated pinwheels. Creation and annihilation rates were confirmed by doubling the number of time frames.

To what extend are the pinwheels of the final pattern just rearrangements of pinwheels at some given time 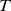
? To answer this question for a given set of pinwheels at an initial time 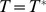 we further calculated the fraction 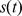 of those pinwheels surviving until time 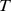. Finally, the fraction of pinwheels present at time 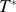 that survive up to the final time 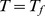 is given by 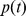.

### Numerical integration scheme

As the Swift-Hohenberg equation is a stiff partial differential equation we used a fully implicit integrator [149]. Such an integration scheme avoids numerical instabilities and enables the use of increasing step sizes when approaching an attractor state. The equation
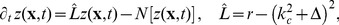
(15)is discretized in time. Using a Crank-Nicolson scheme this differential equation is approximated by the nonlinear difference equation
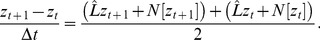
(16)This equation is solved iteratively for 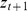 with the help of the Newton method which finds the root of the function
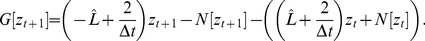
(17)The field 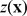 is discretized. For a grid with 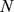 meshpoints in the 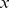-direction and 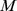 meshpoints in the 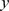-direction this leads to an 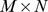 dimensional state vector 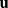. Discretization is performed in Fourier space. The Newton iteration at step 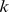 is then given by
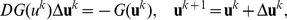
(18)with 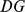 the Jacobian of 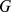. Instead of calculating the matrix 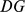 explicitly a matrix free method is used, where the action of the matrix is approximated using finite differences. To solve the linear system 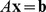 with 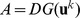, 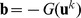 we used the Krylov subspace method [149]. The Krylov subspace of dimensionality 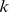 is defined as
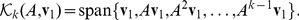
(19)In the *Generalized Minimum Residual* (GMRES) algorithm the Krylov subspace is generated by 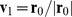 with 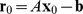, and 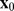 an initial guess, see [149]. After 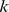 iterations, the refined solution is given by
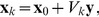
(20)where the matrix 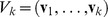 has the base vectors of the Krylov subspace as its columns. The vector 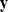 is chosen by minimizing the residuum
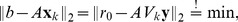
(21)where 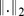 denotes the Euclidean norm. For this procedure an orthonormal basis of the Krylov subspace is generated with an Arnoldi process. With the use of the similarity transformation
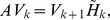
(22)where 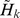 is an upper Hessenberg matrix, 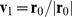, and the orthogonality of 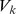, the optimality condition Eq. (21) becomes
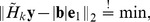
(23)with 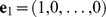 the first unit vector of dimension 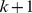. For a 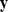 that minimizes this norm the approximate solution is given by 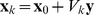. To improve the convergence of this iterative method preconditioning was used. A preconditioner 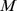 is multiplied to 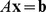 such that 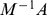 is close to unity. A preconditioner suitable for our model is the inverse of the linear operator in Fourier space with a small shift 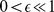 in order to avoid singularities i.e.
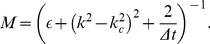
(24)The convergence of Newton's method is only guaranteed from a starting point close enough to a solution. In the integration scheme we use a line search method to ensure also a global convergence [150]. Newton's method Eq. (18) is thus modified as
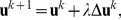
(25)where the function
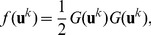
(26)is iteratively minimized with respect to 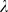.

This integrator was implemented using the *PetSc* library [151]. As the dynamics converges towards an attractor an adaptive stepsize control is very efficient. The employed adaptive stepsize control was implemented as described in [152]. The described integration scheme has been generalized for an arbitrary number of real or complex fields. The coupling terms are treated as additional nonlinearities in 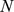. As a common intrinsic timescale we choose 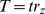 with 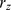 the bifurcation parameter of the OP map. Due to the spatial discretization not all points of the critical circle lie on the grid. Thus, the maximal growth rate on the discretized circle is not exactly equal to 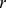, the theoretical growth rate. In particular, some modes may be suppressed or even become unstable. Due to this we expect deviations from analytical solutions. To minimize such deviations the size of the critical circle was chosen such that this disbalance between the active modes was minimized. Periodic boundary conditions were applied to account for the translation invariance of the spatial pattern.

## Supporting Information

Figure S1
**Map interactions with detuned wavelengths and OD stripes.** **C–F** OD stripes interacting with OP columns where 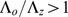. **G** OD stripes interacting with OP columns where 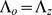. **A,B** Illustration of active modes in Fourier space with 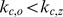, 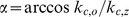. **C,D** 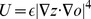, 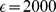, **E–G** 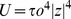, 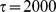, **C,E** 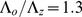, **D,F** 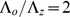. **G** From left to right: initial condition, 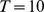, 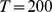, 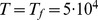. Parameters: 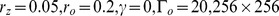 mesh.(TIF)

Figure S2
**Pinwheel nearest neighbor statistics and count variance with detuned wavelengths and OD stripes.** 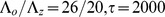 (blue), 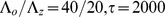 (red), 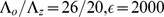 (green), 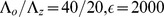 (orange), 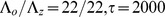 (brown), 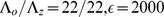 (cyan). **A–F** Mean nearest neighbor pinwheel distance of arbitray **A,B**, equal **C,D**, and opposite charge **E,F**. **G–J** Standard deviation SD of pinwheel density. Shown are the fit parameters for 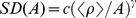. Dashed lines: 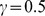. Parameters as in Fig. 9.(TIF)

Figure S3
**Map interactions with detuned wavelength and OD hexagons.** 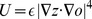. **A** 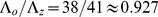, **B** 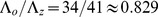, **C** 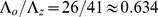, **D** 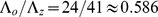, **E** 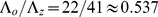, **F** 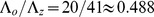. From left to right: initial condition, 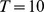, 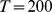, 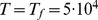. Parameters: 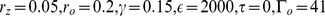, 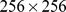 mesh. Initial condition identical in all simulations.(TIF)

Figure S4
**Pinwheel nearest neighbor statistics and count variance with detuned wavelength and OD hexagons.** 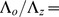 38/41 (blue), 34/41 (red), 26/41 (green), 24/41 (orange), 22/41 (violet), 20/41 (brown), 22/22 (cyan). **A–F** Mean nearest neighbor distance of arbitray **A,B**, equal **C,D**, and opposite charge **E,F**. **G–J** Standard deviation SD of pinwheel density. Shown are the fit parameters for 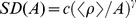. Dashed lines: 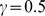. Parameters as in Fig. 11.(TIF)

Figure S5
**Map interactions in higher feature dimensions.** **A,B** Map layout by interactions between three columnar systems (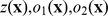). All maps are mutually coupled. Superimposed on the OP map there are the borders of two real fields (black, white). **A** 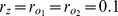 **B** 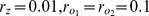. **C,D** Interactions with four columnar systems (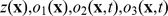). **C** 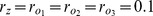. **D** 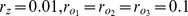. Superimposed on the OP map there are the borders the of three real fields (black, gray, white). From left to right: initial condition, 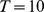, 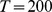, 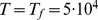. Parameters in all simulations: 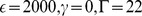, 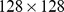 mesh.(TIF)

Figure S6
**Pinwheel nearest neighbor statistics and count variance in higher feature dimensions.** Blue: 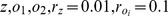, red: 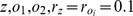, green: 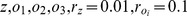, orange: 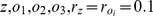. **A–F** Distance to the next pinwheel of arbitrary **A,B**, equal **C,D**, and opposite **E,F** topological charge. **G–J** Standard deviation SD of pinwheel density. Shown are the fit parameters for 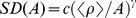. Dashed lines: 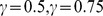. Parameters as in Fig. 13.(TIF)
