# Supplementary figures and images for: Coordinated Optimization of Visual Cortical Maps (II) Numerical Studies
Source: PLoS Comput Biol. 2012 Nov 8;8(11):e1002756. doi: 10.1371/journal.pcbi.1002756 (PMC3493502; doi:10.1371/journal.pcbi.1002756)

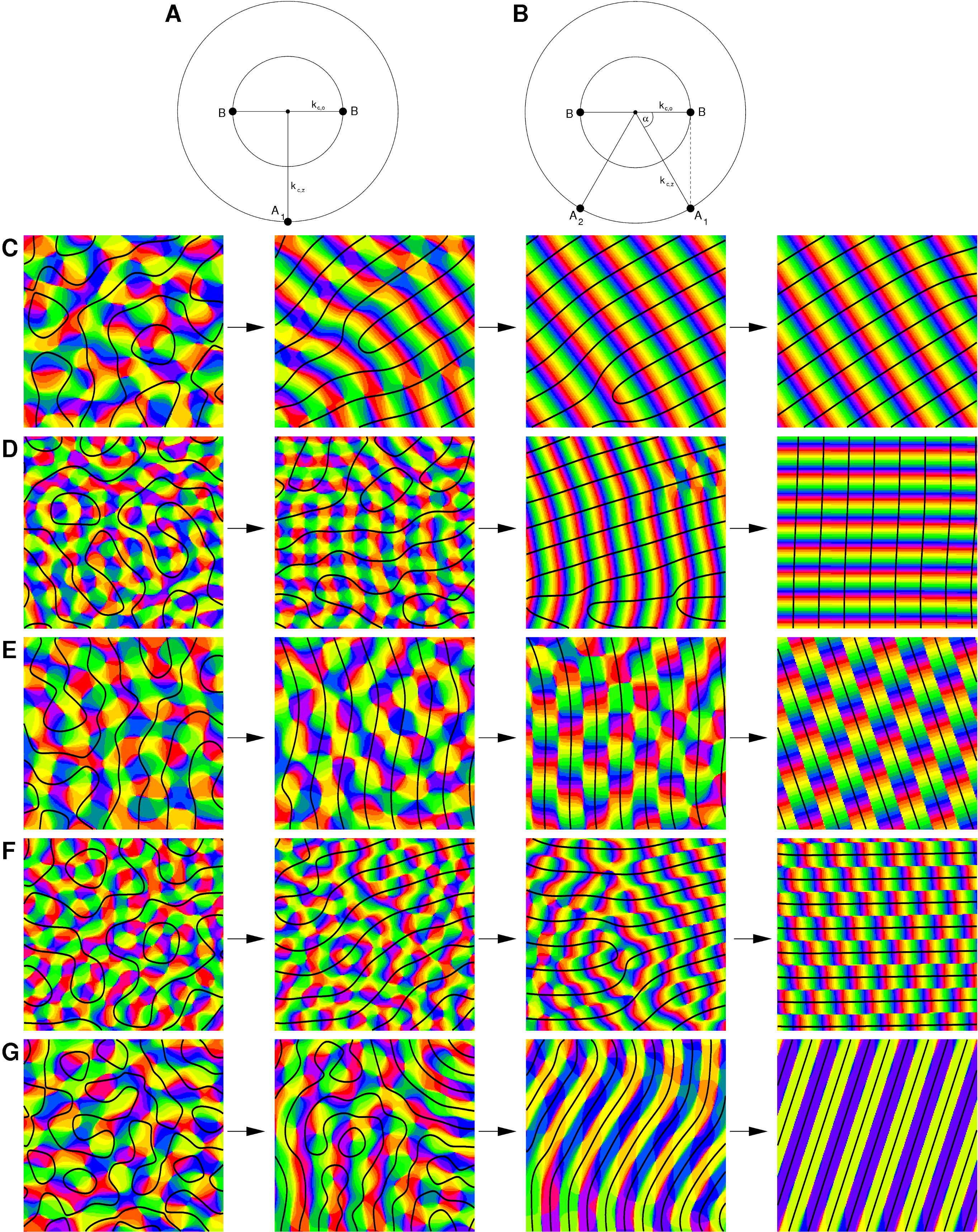

Supplement: Figure S1 — Map interactions with detuned wavelengths and OD stripes. C–F OD stripes interacting with OP columns where . G OD stripes interacting with OP columns where . A,B Illustration of active modes in Fourier space with , . C,D , , E–G , , C,E , D,F . G From left to right: initial condition, , , . Parameters: mesh. (TIF) [file pcbi.1002756.s001.tif]

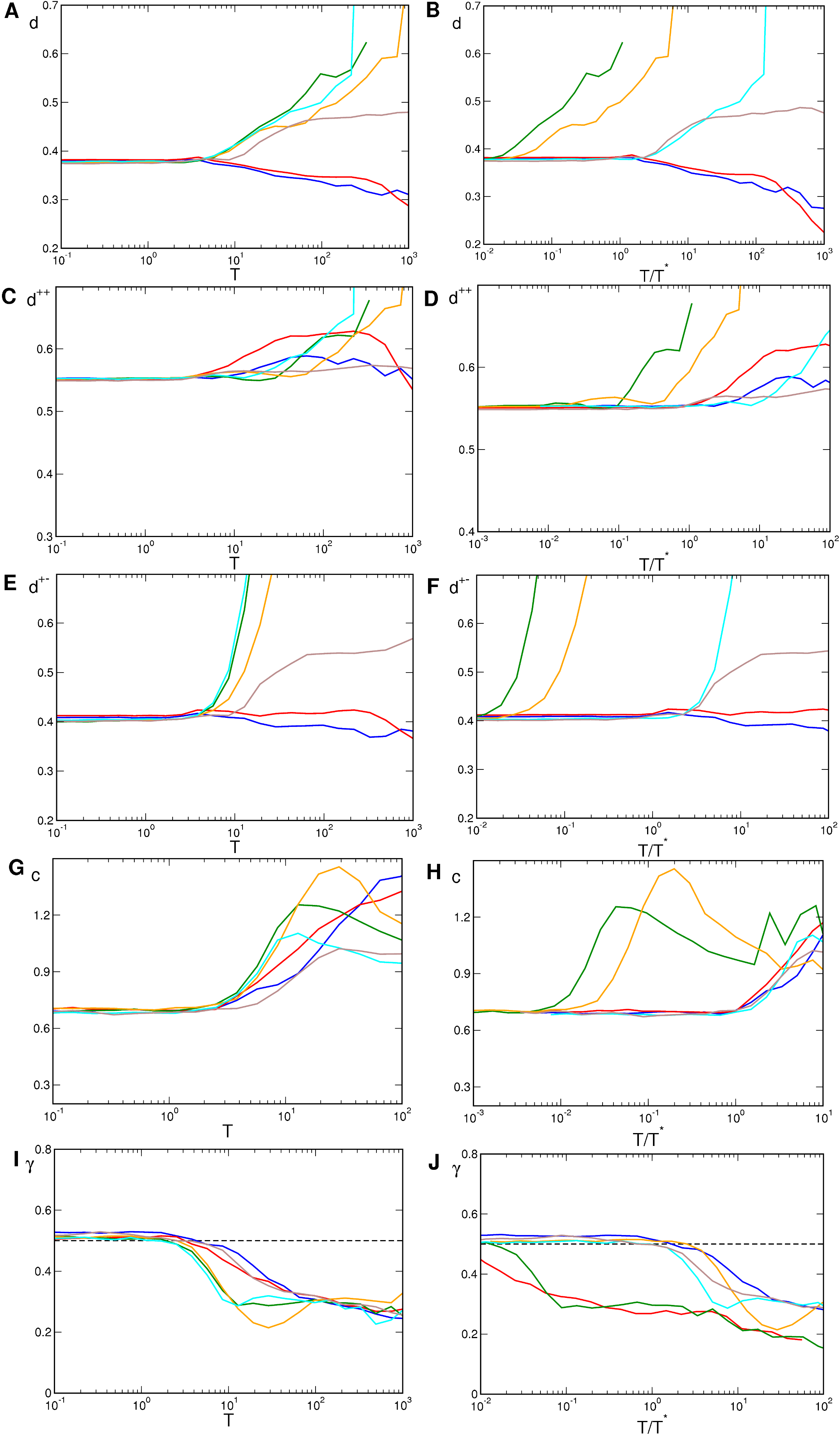

Supplement: Figure S2 — Pinwheel nearest neighbor statistics and count variance with detuned wavelengths and OD stripes. (blue), (red), (green), (orange), (brown), (cyan). A–F Mean nearest neighbor pinwheel distance of arbitray A,B, equal C,D, and opposite charge E,F. G–J Standard deviation SD of pinwheel density. Shown are the fit parameters for . Dashed lines: . Parameters as in Fig. 9. (TIF) [file pcbi.1002756.s002.tif]

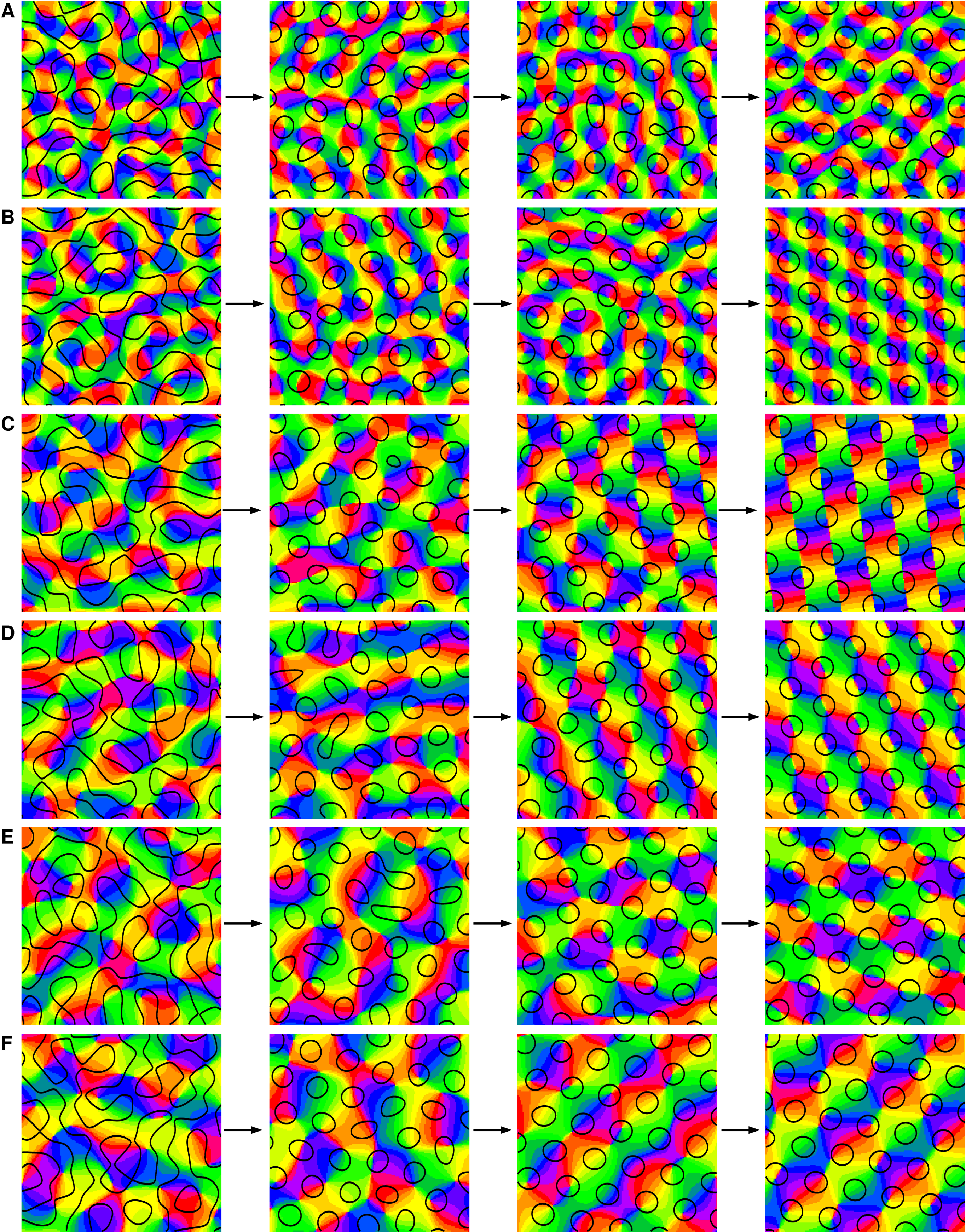

Supplement: Figure S3 — Map interactions with detuned wavelength and OD hexagons. . A , B , C , D , E , F . From left to right: initial condition, , , . Parameters: , mesh. Initial condition identical in all simulations. (TIF) [file pcbi.1002756.s003.tif]

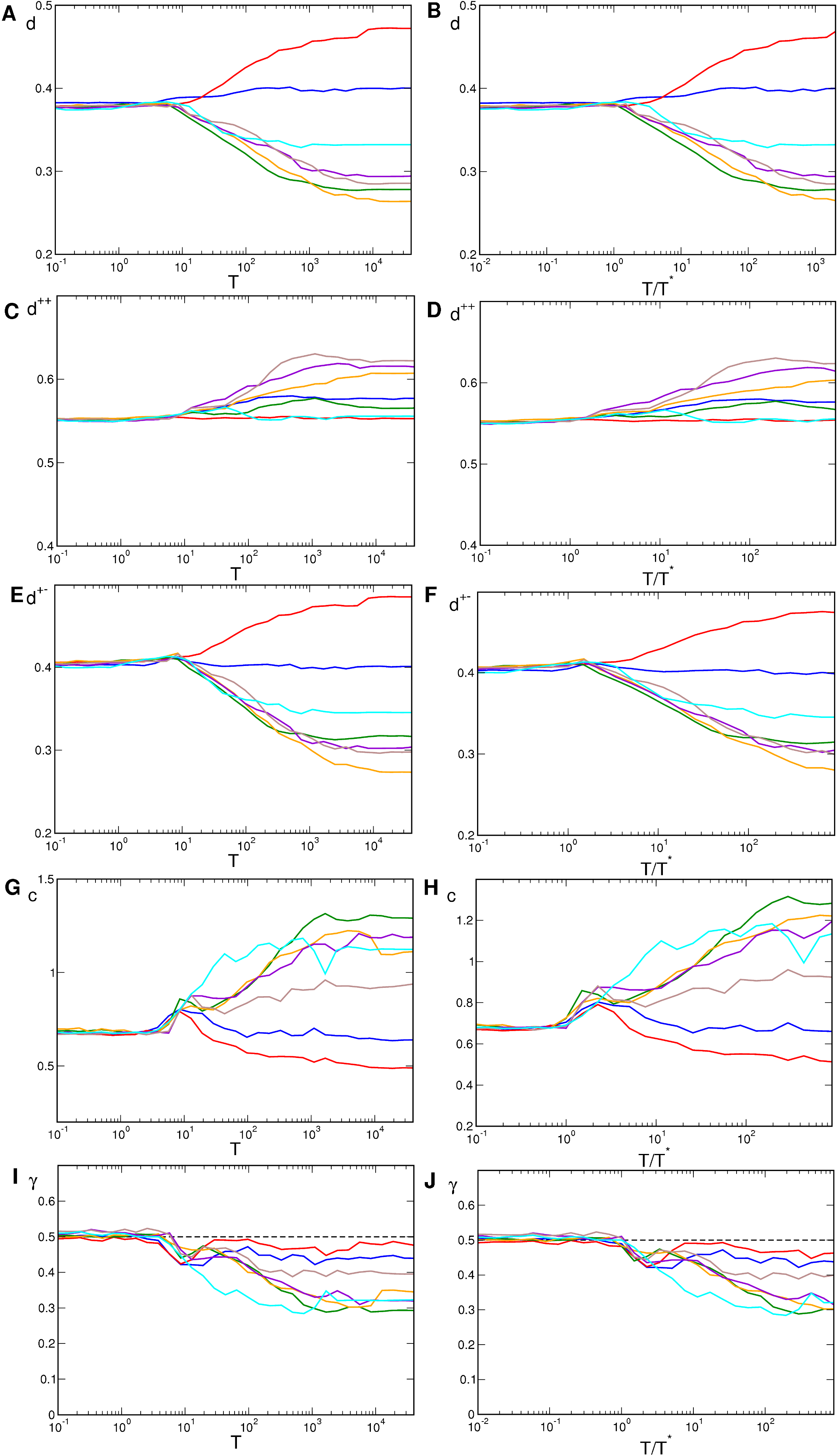

Supplement: Figure S4 — Pinwheel nearest neighbor statistics and count variance with detuned wavelength and OD hexagons. 38/41 (blue), 34/41 (red), 26/41 (green), 24/41 (orange), 22/41 (violet), 20/41 (brown), 22/22 (cyan). A–F Mean nearest neighbor distance of arbitray A,B, equal C,D, and opposite charge E,F. G–J Standard deviation SD of pinwheel density. Shown are the fit parameters for . Dashed lines: . Parameters as in Fig. 11. (TIF) [file pcbi.1002756.s004.tif]

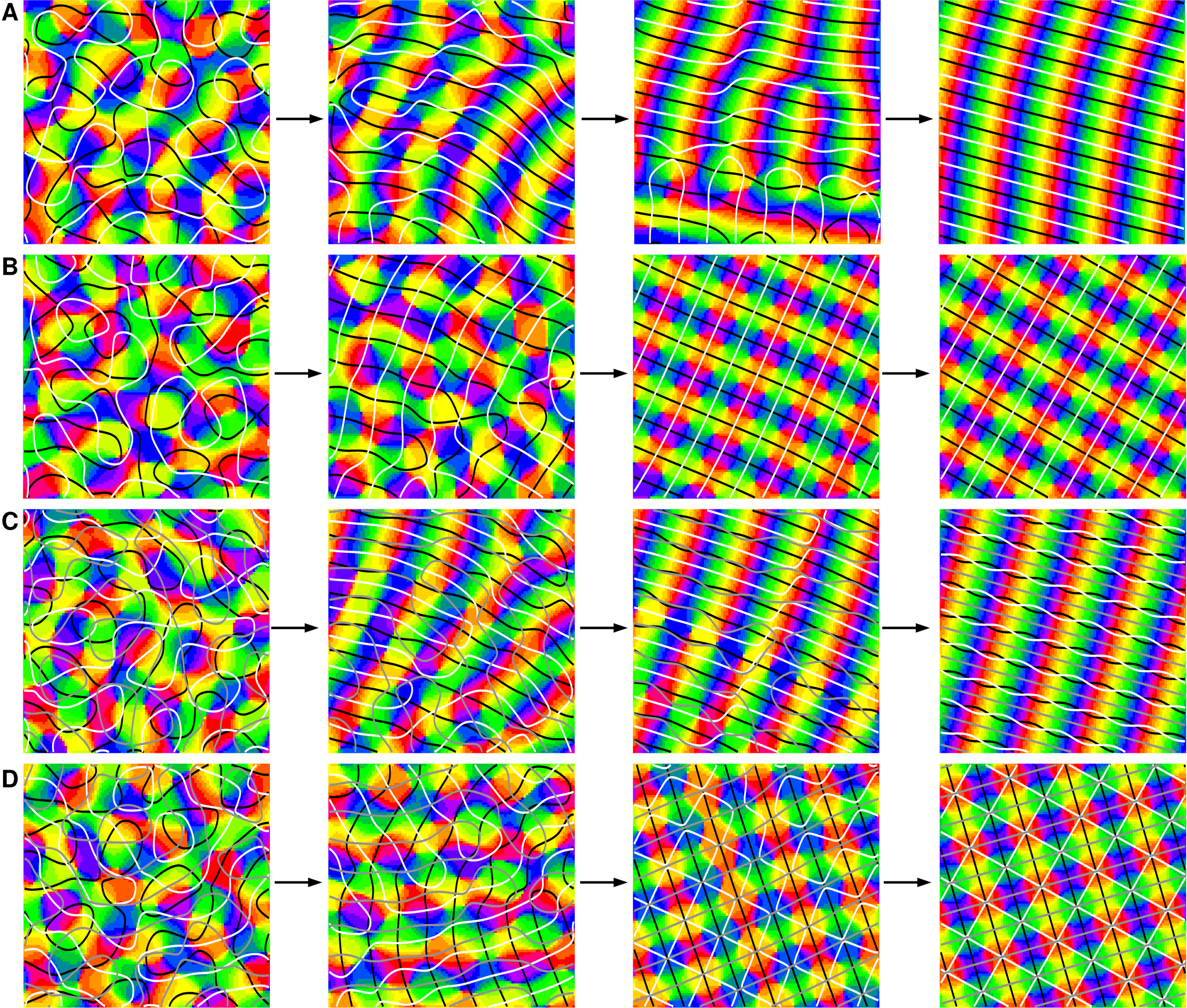

Supplement: Figure S5 — Map interactions in higher feature dimensions. A,B Map layout by interactions between three columnar systems (). All maps are mutually coupled. Superimposed on the OP map there are the borders of two real fields (black, white). A B . C,D Interactions with four columnar systems (). C . D . Superimposed on the OP map there are the borders the of three real fields (black, gray, white). From left to right: initial condition, , , . Parameters in all simulations: , mesh. (TIF) [file pcbi.1002756.s005.tif]

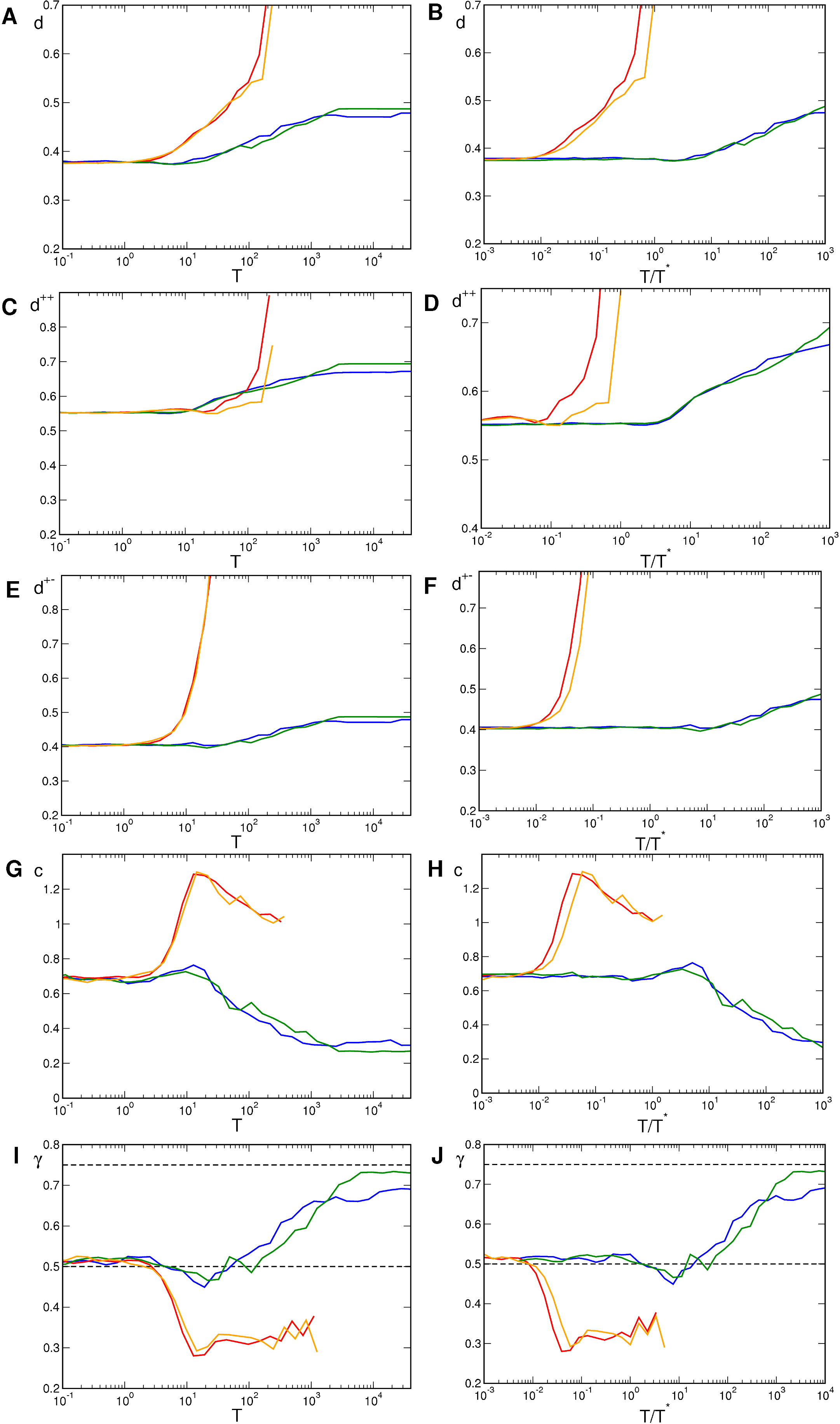

Supplement: Figure S6 — Pinwheel nearest neighbor statistics and count variance in higher feature dimensions. Blue: , red: , green: , orange: . A–F Distance to the next pinwheel of arbitrary A,B, equal C,D, and opposite E,F topological charge. G–J Standard deviation SD of pinwheel density. Shown are the fit parameters for . Dashed lines: . Parameters as in Fig. 13. (TIF) [file pcbi.1002756.s006.tif]
